# Plasma contains ultrashort single-stranded DNA in addition to nucleosomal cell-free DNA

**DOI:** 10.1016/j.isci.2022.104554

**Published:** 2022-06-08

**Authors:** Jordan Cheng, Marco Morselli, Wei-Lun Huang, You Jeong Heo, Thalyta Pinheiro-Ferreira, Feng Li, Fang Wei, David Chia, Yong Kim, Hua-Jun He, Kenneth D. Cole, Wu-Chou Su, Matteo Pellegrini, David T.W. Wong

**Affiliations:** 1 School of Dentistry, University of California, Los Angeles, CA 90095, USA; 2 Department of Molecular, Cellular & Developmental Biology, University of California, Los Angeles, CA 90095, USA; 3 Center of Applied Nanomedicine, National Cheng Kung University, Tainan 704, Taiwan; 4 The Samsung Advanced Institute for Health Sciences & Technology (SAIHST), Samsung Medical Center, Sungkyunkwan University School of Medicine, Seoul 06355, Republic of Korea; 5 Department of Pathology, David Geffen School of Medicine, University of California, Los Angeles, CA 90095, USA; 6 Material Measurement Laboratory, National Institute of Standards and Technology, Gaithersburg, MD 20899, USA

**Keywords:** Biological sciences, Molecular biology, Molecular biology experimental approach, Cell biology, Biological sciences research methodologies, Biology experimental methods

## Abstract

Plasma cell-free DNA is being widely explored as a biomarker for clinical screening. Currently, methods are optimized for the extraction and detection of double-stranded mononucleosomal cell-free DNA of ∼160bp in length. We introduce uscfDNA-seq, a single-stranded cell-free DNA next-generation sequencing pipeline, which bypasses previous limitations to reveal a population of ultrashort single-stranded cell-free DNA in human plasma. This species has a modal size of 50nt and is distinctly separate from mononucleosomal cell-free DNA. Treatment with single-stranded and double-stranded specific nucleases suggests that ultrashort cell-free DNA is primarily single-stranded. It is distributed evenly across chromosomes and has a similar distribution profile over functional elements as the genome, albeit with an enrichment over promoters, exons, and introns, which may be suggestive of a terminal state of genome degradation. The examination of this cfDNA species could reveal new features of cell death pathways or it can be used for cell-free DNA biomarker discovery.

## Highlights

• Plasma contains ultrashort cell-free DNA alongside mononucleosomal cell-free DNA
• The ultrashort cell-free DNA is primarily single-stranded at around 50nt in length
• Ultrashort cell-free DNA sequences are enriched in promoters, exons, and introns

## Graphical abstract

**Figure:**
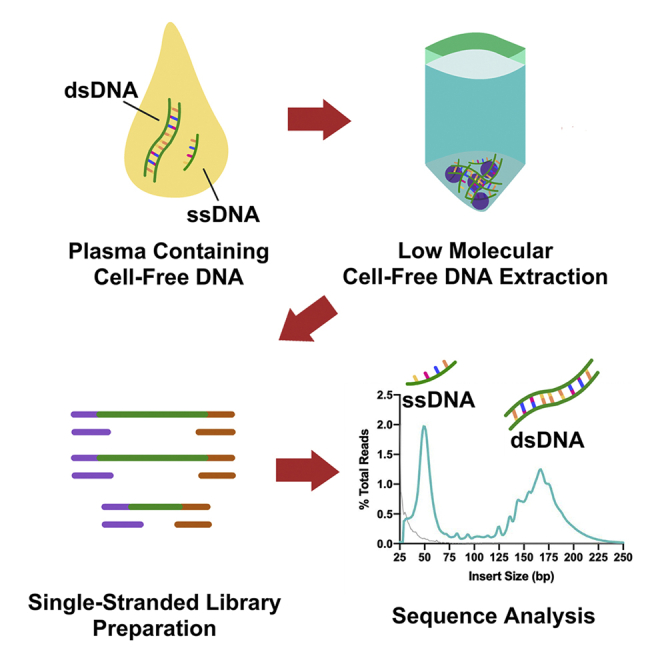

## Introduction

In liquid biopsy, cell-free DNA (cfDNA) analysis is typically focused on the mononucleosomal cfDNA (mncfDNA) biomarker of approximately 160bp in length. However, the current impression of the average fragment length of cfDNA is influenced by the inherent biases of nucleic acid extraction and library preparation. The recent adoption of single-stranded library preparation methods for cfDNA analysis suggests that in addition to mncfDNA, there are shorter cfDNA fragments (<100bp) that can originate from either single-stranded or nicked dsDNA in plasma (Burnham et al., 2016; Snyder et al., 2016). Previous studies indicate that size-selecting for shorter fragments of cfDNA will enrich for mutant-containing cfDNA fragments in late-stage cancer patients (Mouliere and Rosenfeld, 2015). Next-generation sequencing approaches examining whole-genome differences in plasma cfDNA fragment lengths have revealed distinct fragment-profiles in cancer patients compared to those of healthy donors (Cristiano et al., 2019). In addition, groups have attempted to utilize cfDNA strandedness as a diagnostic indicator (Huang et al., 2020; Zhu et al., 2020). With these considerations, ultrashort single-stranded cell-free DNA (uscfDNA) is an unexamined cfDNA entity with potential clinical relevance. In general, nucleic acid extraction kits are not designed to efficiently retain low-molecular cfDNA (<100bp) regardless of strandedness (Diefenbach et al., 2018). Thus, an effective ultrashort ssDNA cfDNA extraction method which retains low-molecular ultrashort cfDNA coupled with single-stranded library preparation could reveal more about cfDNA population in the 25-100 bp (or nt) region.

**Figure 1:**
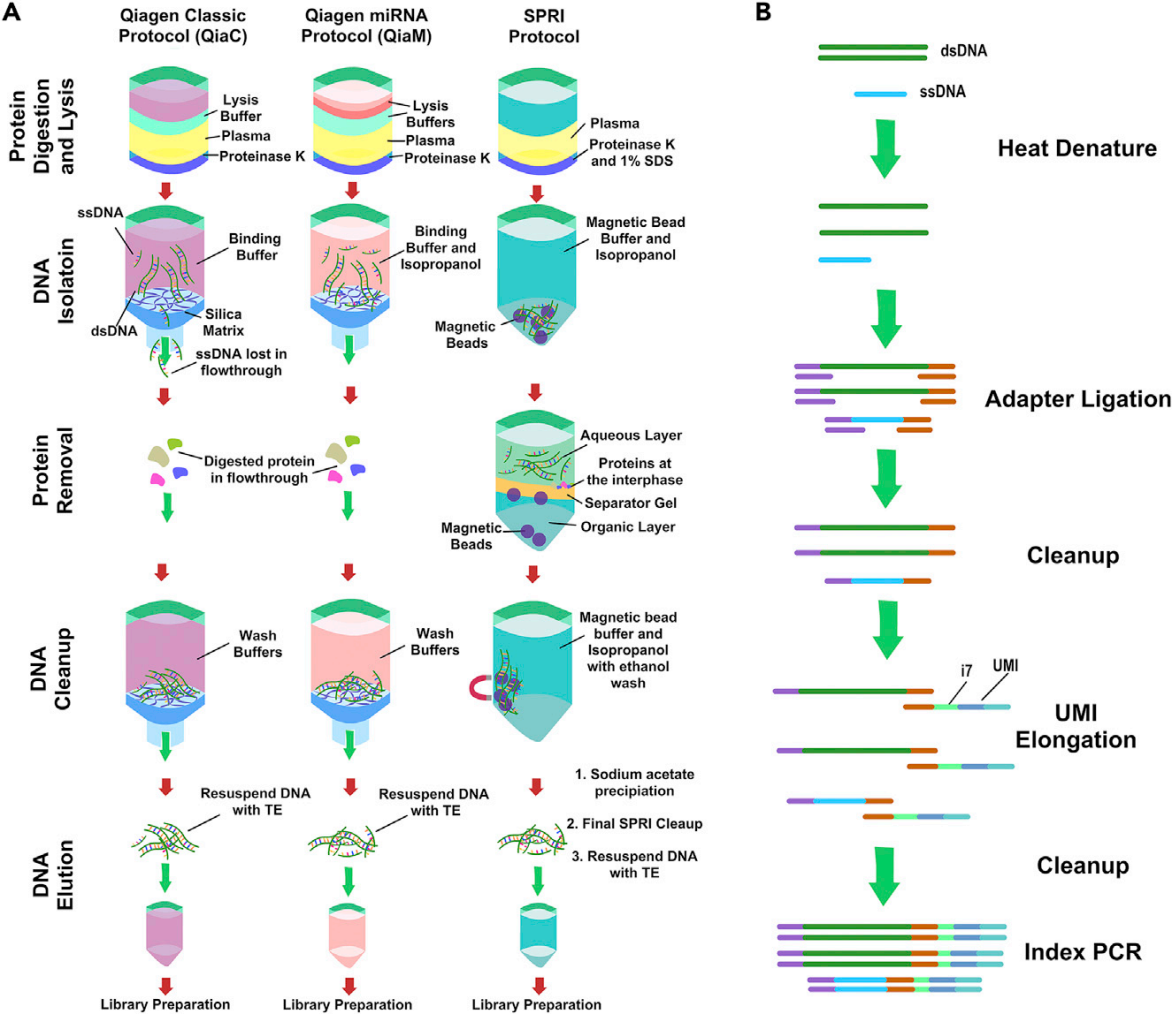
uscfDNA-seq Schematic Protocol (A) Schematic of the three extraction protocols compared in this manuscript. QiaC refers to the QIAGEN QIAamp Circulating Nucleic Acid Kit regular protocol. QiaM refers to the miRNA protocol of the QIAamp Circulating Nucleic Acid Kit. SPRI refers to the (Solid Phase Reversible Immobilization) magnetic beads and phenol:chloroform:isoamyl alcohol protocol. Compared to QiaC, QiaM and SPRI protocols utilize an increased ratio of isopropanol in order to retain the low-molecular nucleic acids for downstream analysis (refer to methods for details). (B) Single-stranded library preparation can incorporate dsDNA, ssDNA, and nicked DNA into the library. Unique molecular identifiers (UMI) are incorporated during the library preparation to remove PCR duplicates.

To address this, we introduce a unique ultrashort single-stranded cfDNA-optimized sequencing pipeline (uscfDNA-seq) (Figures 1A and 1B). This pipeline incorporates an ultrashort single-stranded cfDNA (uscfDNA) extraction method and single-stranded library preparation. The extraction method utilizes both Solid Phase Reversible Immobilization magnetic beads (SPRI) and phenol:chloroform:isoamyl alcohol to retain low molecular weight fragments in plasma. It leverages a high ratio of isopropanol to create a DNA-phobic environment which precipitates out nucleic acids and proteins before isolating the aqueous nucleic acid-containing portion with phenol:chloroform isoamyl alcohol. Subsequent magnetic bead washes help retain the uscfDNA and reduce unwanted contaminants that may affect downstream library preparation enzymes (Figure 1A).

The SPRI extraction method will be compared to two other methods: Firstly, we will include the standard protocol of the commercial silica column-based extraction kit protocol (QIAGEN QIAamp Circulating Nucleic Acid Kit, referred to as QiaC in the manuscript). Secondly, we will compare with the miRNA protocol of the QIAamp Circulating Nucleic Acid Kit, referred to as QiaM, which uses an increased volume in isopropanol, lysis, and binding buffers designed for shorter nucleic acid retention (miRNA).

## Results

### uscfDNA-seq can purify and visualize ultrashort cfDNA in plasma

**Figure 2:**
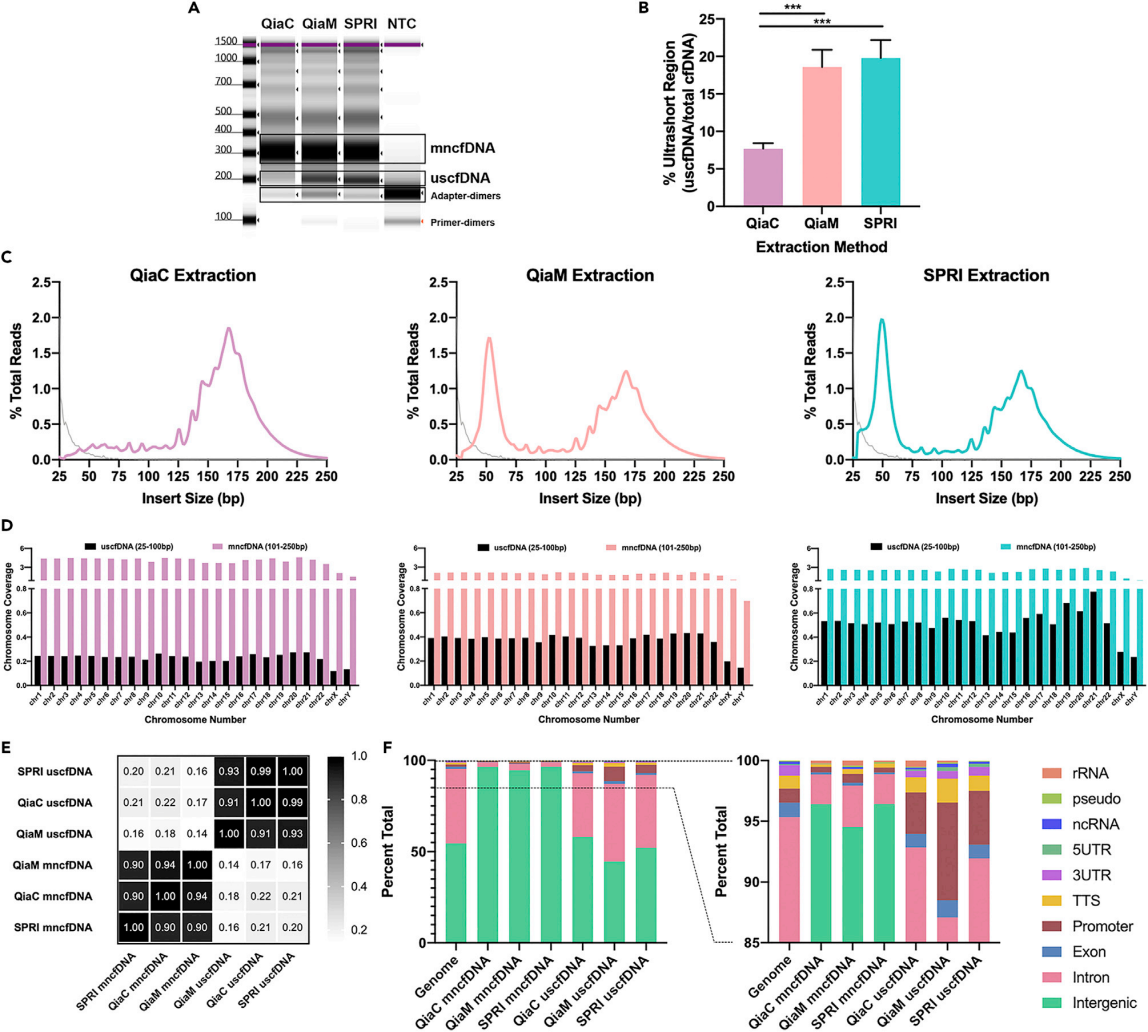
uscfDNA-seq reveals a population of ultrashort cfDNA fragments at 50nt in plasma of healthy donors (A) uscfDNA-seq using QiaM or SPRI reveals a distinct final NGS library uscfDNA band at 200bp (∼50bp after adapter dimer subtraction) compared to QiaC. Electropherogram image was cropped for representative sizes. (B) QiaM and SPRI extraction method can reproducibly isolate the 200bp fragment (180-250bp region in electropherogram) in ten human donors based on quantification of electrophoresis output (200bp band divided by (200bp + 300bp (250-350bp region)). Note: Bands are elongated with ∼150bp of adapters on both sides). ∗∗∗p < 0.001. The paired two-tailed student *t*-test was performed after ANOVA analysis. Bar graphs represent SE of Mean (SEM). See also Figure S2. (C) Alignment of total mapped reads from QiaM and SPRI extracted samples exclusively show the native uscfDNA at 50bp in addition to the mncfDNA peak at ∼160bp when adapters are trimmed. Extraction methods: QiaC (fuchsia), QiaM (pink), and SPRI (teal). Gray line represents sequencing of no template control. (D) The uscfDNA population of the QiaM and SPRI as well as QiaC map along the genome. See also Figure S4. (E) Heatmap of correlation (Pearson) between uscfDNA and mncfDNA coverage of 100bp genome bins for each of the three described methods reveal similarity between the mappings of the uscfDNA and mncfDNA group. (F) Functional group analysis of the reads of mncfDNA and uscfDNA show that the uscfDNA is more similar to the genomic profile. Different extraction methods alter the proportion of function elements. See also Figures S1 and S2.

Single-stranded libraries (Figure 1B) were made from cell-free DNA extracted by QiaM and SPRI methods which revealed a distinct cfDNA band at 200bp in the electropherogram corresponding to about 50bp of insert size (the library preparation adds about 150 bp-worth of adapters) compared to QiaC (Figures 2A and 2B). In all three extraction methods, the mncfDNA peak (300bp before adapter removal) is present.

Similarly, we observed that using the QiaM which incorporates higher isopropanol volume will enhance the capture of low-molecular nucleic acids (Figures 1A and S1A). Interestingly, the miRNA purification protocol is associated with slower flow through the silica column. SEM images of the silica column indicate a reduction in pore size accompanied by sheet-like deposits possibly derived from increased isopropanol precipitation of organic matter in the plasma (Figure S1B). As part of uscfDNA-seq these two extraction methods optimized for short DNA are partnered with a single-stranded library construction in order to fully visualize and examine the cfDNA population that is smaller than 100bp.

In a supplemental experiment, we used the QiaC protocol with centrifuge (as opposed to vacuum) in order to collect the flow through of the binding step of the standard QiaC protocol for the presence of low-molecular weight DNA. The QiaC flow through was subsequently extracted with QiaM (with increased isopropanol and lysis and binding buffers) to reveal that the uscfDNA could be rescued (Figure S1C). This also indicates that the QiaC protocol has a tendency to lose low-molecular DNA.

### uscfDNA is consistently present in plasma independent of blood collection methods

This is a reproducible phenomenon with similar observations in multiple donors (Figures 2B and S2A). Although we have shown that plasma from K2EDTA vacu-containers contain uscfDNA (Figure 2), K2EDTA tubes are often reported to be associated with cell-free DNA degradation (Parpart-Li et al., 2017). Thus, to rule out the possibility of uscfDNA as an artifact of sample collection, StreckDNA tubes (the gold-standard for cell-free DNA preservation due to their ability to decrease white blood cell rupture and subsequent genomic DNA contamination in the sample) was also tested for presence of uscfDNA. An alternative, StreckRNA, which is used to preserve RNA (a low molecular nucleic acid) and exosomes was also tested. All three collection tubes allowed us to detect the presence of the uscfDNA population (Figure S2B). Extractions performed from the TE buffer alone did not manifest any uscfDNA or mncfDNA bands except for adapter-dimer bands introduced by the library preparation protocol (Figure S2C). In addition, treatment with RNase Cocktail digestion before library preparation did not appreciably decrease the uscfDNA band ruling out the presence of RNA (Figure S1D).

### Magnetic bead extraction methods may capture short and single-stranded DNA molecules better than silica column-based methods

To compare the efficiency of the extraction methods, nonhuman ssDNA oligos designed from the *Escherichia coli* phage lambda genome of sizes 30, 50, 75, 100, 150, and 200nt (Table S2 and STAR Methods) were spiked into the plasma prior to extraction and library preparation. The uscfDNA extraction methods (QiaM and SPRI) retain ultrashort fragments in plasma with greater efficiency compared to the regular QiaC protocol (Figures S3A and S3B). Interestingly, the SPRI extraction method showed improved retention of 30 and 50nt ssDNA compared to QiaM. Although these two extraction methods show improved ability in retaining low-molecular ssDNA, their yield suggests that there is still substantial loss. Hence, further refining of future methods to improve the yield is warranted. Advantages of the current bead-based methods are that they limit physical loss of ultrashort cfDNA fragments compared to silica columns that utilize flow through the pores. However, the observed presence of adapter-dimers is suggestive of the presence of inhibiting factors in SPRI derived cfDNA products that may interfere with downstream enzyme activity.

### uscfDNA reads map evenly and predominantly to nuclear human DNA sequences

Upon sequencing and alignment to the human genome, the reads were divided into two distinct size populations (25-100bp named uscfDNA and 101-250bp named mncfDNA) with QiaM and SPRI both showing increased coverage of the ultrashort population (Figure 2C). The reads corresponding to the ultrashort population are evenly distributed across the genome, although SPRI-extracted uscfDNA shows some increase in chromosomes 19 and 21(Figure 2D). It has been previously reported that mitochondria-derived cell-free DNA is fairly short (50bp) but we found that it only contributed a minority (<0.1%) of the total mappable DNA reads (Figure S4A). QiaM and SPRI are enriched for mitochondrial DNA in the uscfDNA population but still are a minor fraction of total DNA (Figure S4B). Examining the correlation of the mapping between uscfDNA and mncfDNA extracted with the three methods revealed consistent homogeneity within the uscfDNA and mncfDNA populations (Figure 2E).

### The functional element ratio of uscfDNA sequences resembles that of the genome

We examined the functional elements profile of the mncfDNA and uscfDNA sequences amongst different extraction methods to identify any characteristic patterns (Figure 2F). Compared to the genomic distribution of the functional elements, the mncfDNA profile presented an increased enrichment in the intergenic sequences and marked decrease in introns, exons, and promoters. In contrast, the uscfDNA more closely resembled the genome but had a noted increase in promoter, exon, and intron sequences. Between extraction methods, the QiaM-extracted uscfDNA had the greatest proportion of promoter regions mapping compared to QiaC and SPRI-extracted uscfDNA.

### uscfDNA is predominantly single-stranded

**Figure 3:**
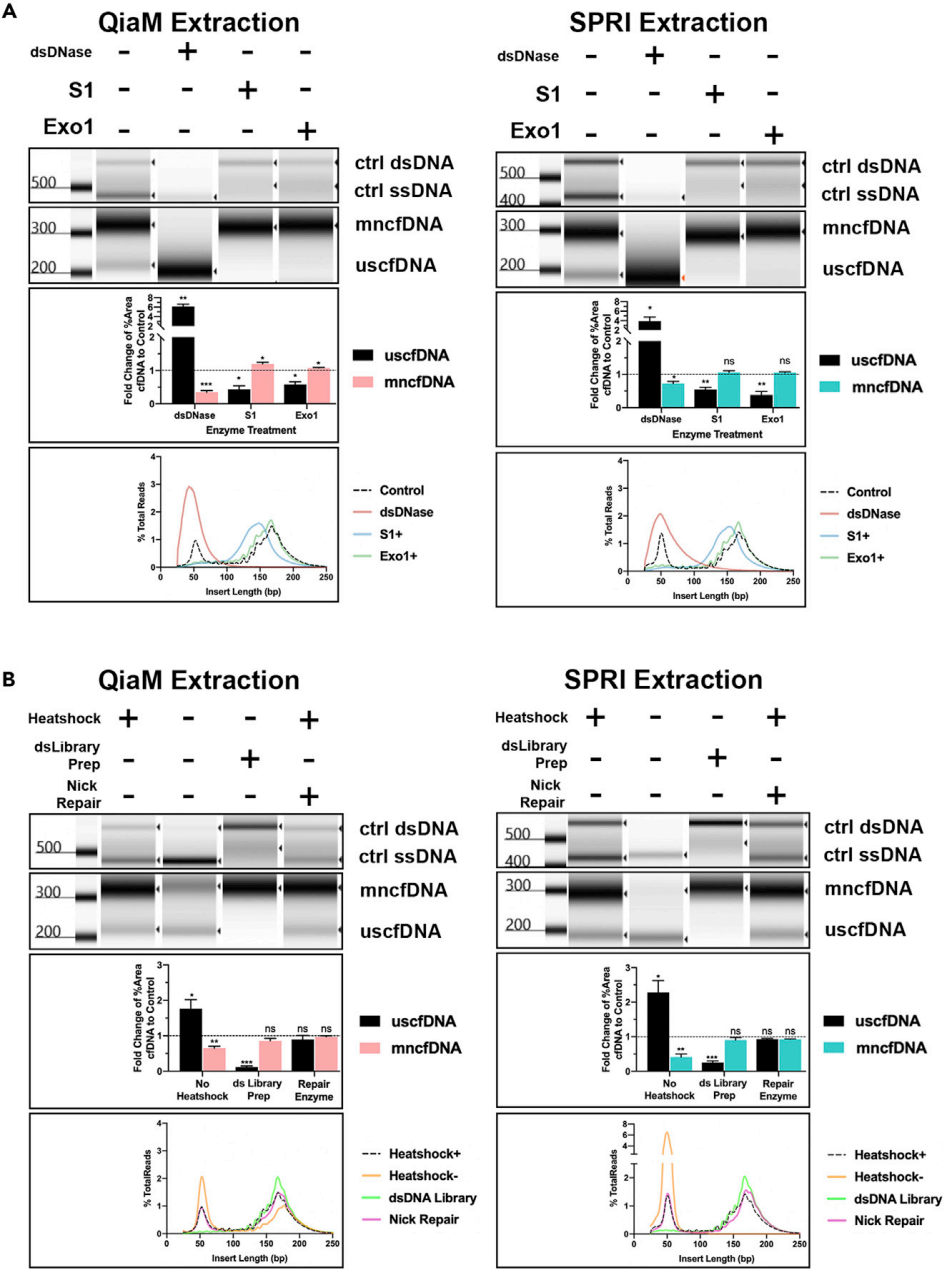
uscfDNA population is predominantly single-stranded (A) Size distribution of final library digestion with cfDNA supplemented with control oligos. (B) Size distribution of library preparation variations with cfDNA supplemented with control oligos. For A and B, Top panels: electrophoretic visualization. Middle panels: quantification of the mapped reads belonging to the short (uscfDNA) or long population (mncfDNA). Bottom panels: mapped read size distribution. Reads with insert size under 25bp and above 250 were excluded from the plots. Bar graphs composed of plasma from three different human donors. The paired two-tailed student *t*-test was performed after ANOVA analysis. ∗p < 0.05, ∗∗p < 0.01, and ∗∗∗p < 0.001. Sequences from the lambda genome of 460bp dsDNA and 356nt ssDNA were used as positive controls. Adapter-dimers have been cropped from the presented electropherograms. Bar graphs represent SE of Mean (SEM). Electropherogram images were cropped for representative sizes. See also Figures S5 and S6.

To examine the properties of strandedness, the extracted cfDNA supplemented with two control oligos (250 nt single-stranded and 350 bp double-stranded) was subject to strand-specific enzymes. When the DNA extracts were subject to dsDNA-specific DNase (dsDNase) digestion, the mncfDNA (300 bp) and the control dsDNA bands (500 + bp) showed a clear reduction in intensity as evidenced by the electrophoresis of the corresponding final libraries (Figures 3A and S5A). In contrast, digestion by single-strand specific nucleases (S1, Exo 1, and P1) showed significant reduction in the uscfDNA band and the control ssDNA band (400 + bp) while preserving the mncfDNA band and the control dsDNA band (500 + bp) in plasma extracted by both the QiaM and SPRI protocols. Sequencing and alignment of these libraries confirmed the results from the electropherograms (Figure 3A, bottom panels). These results strongly indicate the single-stranded nature of the uscfDNA.

To corroborate the single-stranded nature of this DNA we leveraged the differences in the adapter ligation chemistry between ssDNA and dsDNA library kits (Figure 3B). The uscfDNA peak was absent in the dsDNA library preparation (which only processes intact double-stranded substrates) suggesting that the ultrashort population is endogenously single-stranded in nature. By contrast, the ssDNA library kits require initial heat denaturation (98°C for 3 min) to efficiently incorporate dsDNA molecules into the library. By skipping this step, the presence of the 200bp population remained suggesting that the uscfDNA population is mostly single-stranded (Figure 3B). Finally, to determine if the source of the uscfDNA derived from nicked dsDNA, we pretreated the extracted nucleic acids with a nick repair enzyme but did not observe a reduction of ultrashort fragments in the final library. This suggests that the vast majority of uscfDNA are not derived from nicked mncfDNA. These observations were consistent among three replicates (Figures S5A and S5B).

Alignment of sequenced digestion libraries recapitulated the findings previously mentioned with some interesting observations (Figures 3A, 3B, S6A, and S6B). Firstly, the S1 treated samples showed a 10bp downshift in the modality of the mncfDNA peak (from 160 to 150bp). Secondly, both the S1 and nick-repair enzyme treatment flattened the periodicity on the left side of the mncfDNA peak. These observations suggest that the 10bp periodicity may be a result of nicked mncfDNA at certain fragment lengths. The S1 enzyme may also be digesting jagged edges flanking the mncfDNA. Heatmap correlation of the digestions show that in both QiaM and SPRI extraction methods, the mncfDNA and uscfDNA populations group together (Figures S7A and S7B).

### Functional element analysis of digested samples corroborates with that uscfDNA has an increased proportion of promoter, intron, and exon regions compared to genome

**Figure 4:**
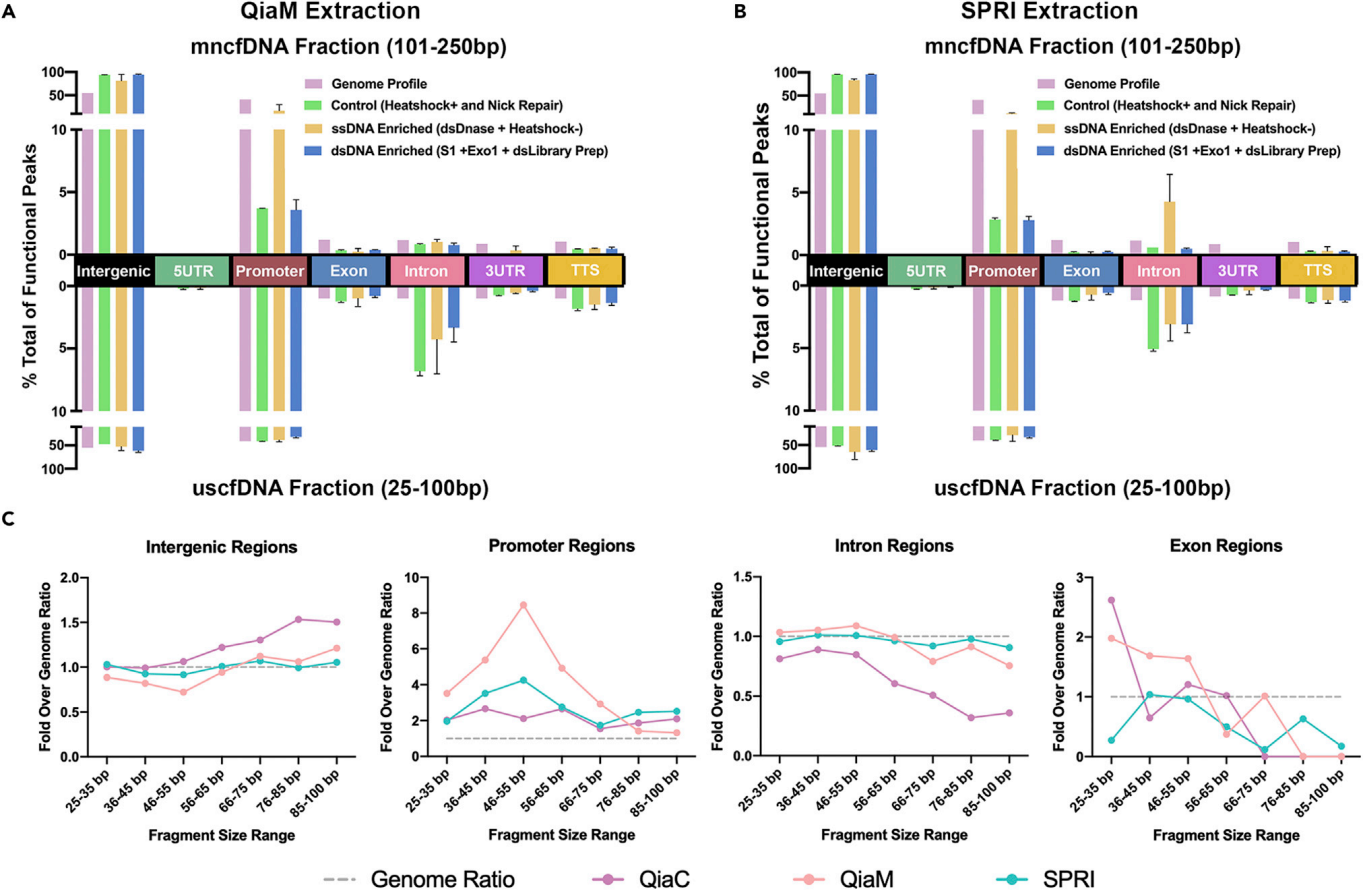
Enrichment of mncfDNA or uscfDNA using pre-library digestions reveal their functional characteristics Summation of ssDNA enrichment treatments (dsDnase and Heat shock) and dsDNA enrichment (S1, exo 1, and dsLibrary preparation) present different function peak profiles in the mncfDNA and uscfDNA fraction along different elements of a typical gene in samples extracted by (A) QiaM and (B) SPRI. (C) Compared to the genome ratio (gray dotted line) the proportion of functional peaks varies at different uscfDNA fragment sizes. Different patterns are observed in different extraction methods. Bar graphs represent SE of Mean (SEM). See also Figures S7 and S8.

We attempted to use the functional element peak profiles (Figures S7C and S7D) from the QiaM and SPRI digestions to see if they could generalize the functional characteristics differences in mncfDNA and uscfDNA observed earlier (Figure 2F). By summating dsDNase and non-heat shock treatments to model uscfDNA enrichment and S1 nuclease, exo one nuclease, and dsDNA library preparation to model mncfDNA enrichment, we recipulated that uscfDNA is elevated in promoters, exons, and introns where mnfDNA is elevated in intergenic regions (Figures 4A and 4B). Regardless, independent treatments revealed some unique findings. When samples were treated with dsDNase, the mncfDNA fraction appeared to mimic the uscfDNA (of untreated samples) in regards to increased promoter, exon, and intron fractions accompanied with a lowered intergenic localization. It initially appeared counter intuitive that dsDNase (which should reduce the mncfDNA) lead to a decrease in promoter and exon fraction in the uscfDNA fraction, but it may be because of degraded mncfDNA fragments flooding the uscfDNA size pool. Mirroring this, treatment with dsDNA library preparation led the uscfDNA fraction to mimic the mncfDNA by decreasing the promoter and exon ratio and increasing the intergenic regions.

### The proportion of functional peaks vary at different uscfDNA fragment sizes

We further divided the uscfDNA population in 10bp-sized intervals to test whether there was an association between functional peak proportion and specific fragment sizes (Figures 4C and S8). In both QiaM and SPRI extraction methods there was a clear increase of promoter regions in sequences sized 45-55bp compared to the genome and the QiaC extraction method. Similarly, a small increase occurred for introns and exons at 35–45 and 45-55bp. Interestingly, the intergenic regions proportion increased steadily as the sequences got closer to 100bp for all three extraction methods. Compared to QiaM and SPRI, QiaC behaved more sporadically due to having fewer total reads (12.2 (QiaC) vs 23.2 (QiaM) and 31.2 (SPRI) million) the 25-100bp region to begin with (Table S4).

## Discussion

The revelation that there are two distinct populations of cfDNA opens up several new avenues for scientific exploration. Firstly, the field of molecular diagnostics must now consider the uscfDNA population, in conjunction with conventional mncfDNA, for biomarker identification and diagnosis. Therefore, in liquid biopsy for cancer detection, uscfDNA could provide a new resource of available biomarkers. It has long been observed that in late-stage cancer, not only does the concentration of cell-free DNA increase, the average fragment length also decreases by 10-20bp(Lapin et al., 2018). Mutation containing cell-free DNA is consistently shorter than wild-type DNA and this skewed impression fragment size in late-stage cancer is likely because of the increased ratio of cancer cells undergoing apoptosis (Mouliere et al., 2018). These previous studies, however, only utilize extraction and DNA-quantification methods that consider the double-stranded mncfDNA population. Whether this observed pattern in late-stage cancer donors is mirrored by uscfDNA is not clear. Conversely, a study on cfDNA from pancreatic patient plasma using single-stranded library preparation (extracted with the equivalent of QiaC) showed that earlier stages are actually associated with shorter fragments (Liu et al., 2019). This apparent contradiction may hint that size profiles and concentrations of these two populations of cfDNA may have contrasting trajectories between the healthy, early-stage, and late-stage cancer phases.

Because the uscfDNA has enriched promoter, exon, and intron elements compared with the mncfDNA (Figures 2F, 4A, and 4B), uscfDNA could be a better reservoir for specific biomarker sequences. Most genetic aberrations in diseases are associated with coding regions and not the intergenic sequences enriched in mncfDNA. There may be merit in using single-stranded library preparation kits without the initial heat shock if investigators wish to enrich uscfDNA fragments in their final library. Although in theory, dsDNase treatment should enrich the library for uscfDNA, it actually lowers the percent of promoters, introns, and exons by possibly adding degraded mncfDNA molecules to the uscfDNA size pool (Figures 4A and 4B).

When looking for rare mutations, the short footprint of uscfDNA should be considered for calculations regarding genomic coverage. Because of uscfDNA having shorter reads, libraries with substantial uscfDNA population will require more total reads to achieve the same genomic coverage as a mncfDNA dominant library (Figure 2D and Table S4) (Desai et al., 2013). Therefore, target capture to enrich the coverage in certain regions will be required for any rare mutation detection. By applying target-capture enrichment, we have previously found evidence that ultrashort circulating tumor DNA contained in plasma from non-small cell lung carcinoma patients can also harbor mutations corresponding to the mncfDNA and tissue genotyping (Li et al., 2020). However, in contrast to the methodology we present here, the pipeline was not optimized for single-strand DNA. By incorporating this uscfDNA-seq methodology, we are actively exploring how uscfDNA fragment patterns are altered in different disease states in clinically-focused studies.

Secondly, uscfDNA introduces new potential biological insights in cfDNA biology. The functions of RNA, a prominent single-stranded entity, are well described. RNA is involved in transcription, amino acid transfer, protein-complexes, gene expression, and signal-transfer via exosomes. By comparison, circulating ssDNA biology has been largely unexplored, and it is plausible that ssDNA may have more functions than initially thought. In molecular biology, there is limited technology to evaluate ssDNA. With the development of uscfDNA-seq, future studies interested in the assessment of ultrashort single-stranded DNA molecules is possible. In this regard, there is merit in exploring how uscfDNA plays a role in normal physiology and how it may change with age in comparison to the mncfDNA population (Teo et al., 2019).

In regards to its origins, based on the data presented here, uscfDNA appears to be involved in the cell death pathways for the disposal of genomic DNA. Extensive literature has described the origins of mncfDNA as a byproduct of genomic DNA degradation (Burnham et al., 2016; Nagata et al., 2003). Based on our observations, the genomic coverage of uscfDNA maps evenly amongst the chromosomes in the genome mirroring the pattern of mncfDNA (Figure 2D). However, examination of the function elements of uscfDNA provides additional insights since uscfDNA closer resembled the genomic profile but with a marked enrichment in promoter sequences (Figure 4C) at 50nt. The observed enrichment may be suggestive of originating from transcription factor-bound complexes to one strand of DNA (Tomonaga and Levens, 1996). In contrast, the mncfDNA fragments had an observed decrease in exon, intron, and promoter sequences. These coding regions would be expected to be accessible for active transcription and susceptible to initial nuclease degradation unlike the nucleosomal-protected intergenic sequences. Therefore, uscfDNA could be derived from both exposed regions of the genome and eventual metabolism of nucleosome-protected mncfDNA. Recent work has begun describing possible nucleases such as DNase1, DNASE1L3, and DFFB that contribute to the regulation of mncfDNA processing (Han et al., 2020). Since uscfDNA-seq can now readily detect and analyze uscfDNA in biological samples, it is paramount to explore the nucleases which regulate its appearance in blood.

Aside from part of a degradation pathway it is plausible that that uscfDNA could be involved in biological processes. Although not yet described in eukaryotes, the bacterial genome contain “retrons” sequences which code for a special type of reverse transcriptase and a noncoding RNA sequence to generate DNA/RNA hybrid called multicopy single-stranded DNA (msDNA)(Inouye and Inouye, 1993; Schubert et al., 2021). The retron ssDNA thought to be part of the bacterial immune system and helps to detect for invading viruses (Millman et al., 2020). Some msDNA have been described to be as short as 48 nt so it is conceivable that an eukaryotic version may contribute to the uscfDNA pool in plasma where the RNA component has already degraded (Mao et al., 1997).

In addition, further work must be performed to evaluate the impact of extraction methods and the downstream recovery of uscfDNA. Based on the functional peak analysis it appears although QiaM and SPRI can recover uscfDNA in plasma, they may be recovering a different population profile (Figures 2F and 4A–4C). It appears that QiaM may be enriched for promoter and exon sequences (which may be important for disease detection) but size efficiency experiments indicate that SPRI has greater recovery of 30-50nt uscfDNA (Figure S3B). However, sequences shorter than 50bp may have greater intergenic proportion which would result in the dilution of sequences in coding regions for SPRI extracted samples (Figure 4C). During our manuscript revisions we discovered that three other research groups (one unpublished) also isolated ultrashort single-strand fragments in plasma using distinctly different methods (Cheng et al., 2021; Hisano et al., 2021; Hudecova et al., 2021). There would be interest to cross-compare these methods in future studies.

In conclusion, we demonstrate the uscfDNA-seq pipeline (which can use two cell-free DNA extraction methods, one modified commercial and one customized, with a single-stranded library preparation) reveals the presence of a unique class of ultrashort single-stranded cell-free DNA of nuclear origin with a modal size of 50nt. Careful examination of uscfDNA may likely provide new opportunities in molecular diagnostics and cfDNA biology in the future.

### Limitations of the study

Limitations of this study include unequal male and female ratio of subjects with an average age of 32. In addition, functional element analysis was derived from the sequenced samples from a single donor.

## STAR★Methods

### Key resources table

**Table:** 

REAGENT or RESOURCE	SOURCE	IDENTIFIER
**Biological samples**		

Single Donor Human Plasma for Research	Innovative Research	IPLASK2E2ML

**Chemicals, peptides, and recombinant proteins**		

Proteinase K (20 mg/mL)	Zymogen	D3001-2-125
TE Buffer (pH 8.0, 0.2uM filtered, 10mM Tris, 1mM EDTA)	Invitrogen	AM9849
Isopropanol	Fisher	BP26181
Phase Lock Tubes (Heavy)	Quantabio	10847-802
Phenol:Chloroform:Isoamyl Alcohol 25:24:1 Saturated with 10 mM Tris, pH 8.0, 1 mM EDTA with equilibrium buffer.	Sigma	P2069-100ML
SPRI Beads	Beckman Coulter	B23318
Glycogen (20 mg/mL)	ThermoFisher	R0561
Sodium Acetate (3M)	Quality Biological Inc	50-751-7660
DNase I	Invitrogen	18-068-015
S1	ThermoFisher	EN0321
EDTA	ThermoFisher	15575020
P1	NEB	M0660S
Exo I	NEB	M0293S
dsDNase	ArcticZyme	70600-201
PrePCR Repair	NEB	M0309S
RNase Cocktail	ThermoFisher	AM228

**Critical commercial assays**		

QIAamp Circulating Nucleic Acid Kit	QIAGEN	55114
Buffer ATL	QIAGEN	939011
SRSLYTM PicoPlus DNA NGS Library Preparation Base Kit, 24 rxn	ClaretBio	CBS-K250B-24
SRSLYTM 12 UMI-UDI Primer Set, 24 rxn	ClaretBio	CBS-UM-24
SRSLYTM UMI Add-On Reagents, 24 rxn	ClaretBio	CBS-UR-24
SRSLYTM Clarefy Purification Beads, 24 rxn	ClaretBio	CBS-CB-24
NEBNext® Ultra™ II DNA Library Prep Kit for Illumina	NEB	E7645S
Unique Dual Index UMI Adapters RNA Set 1	NEB	E7416S
MyTaq HS Mix	Bioline	BIO-25045
Qubit™ Fluorometer	ThermoFisher	Q33327
Qubit™ dsDNA HS and BR Assay Kits	ThermoFisher	Q32851
Qubit™ Assay Tubes	ThermoFisher	Q32856
TapeStation 4200	Agilent	G2991BA
TapeStation Tapes HSD1000	Agilent	5067-5584
Loading Tips	Agilent	5067-5153
High Sensitivity D1000 Reagents (Ladder and Buffer)	Agilent	5067-5585
Optical tube strip caps (8x Strip)	Agilent	401425
Optical tube strips (8x Strip)	Agilent	401,428

**Deposited data**		

Raw and analyzed data	This paper	GEO: GSE202433

**Oligonucleotides**		

Lambda dsDNA Control - 5’-CAAACTGCGCAACTCGTGAA AGGTAGGCGGATCCCCTTCGAAGGAAAGACCTGATGCT TTTCGTGCGCGCATAAAATACCTTGATACTGTGCCGGA TGAAAGCGGTTCGCGACGAGTAGATGCAATTATGGTT TCTCCGCCAAGAATCTCTTTGCATTTATCAAGTGTTTCCT TCATTGATATTCCGAGAGCATCAATATGCAATGCTGTT GGGATGGCAATTTTTACGCCTGTTTTGCTTTGCTCGAC ATAAAGATATCCATCTACGATATCAGACCACTTCATTTC GCATAAATCACCAACTCGTTGCCCGGTAACAACAGC CAGTTCCATTGCAAGTCTGAGCCAACATGGTGATGAT TCTGCTGCTTGATAAATTTTCAGGTATTCGTCAGCCGT AAGTCTTGATCTCCTTACCTCTGATTTTGCTGCGCGAGT GGCAGCGACATGGTTTGTTGT-3’	IDT	N/A
Lambda ssDNA Control - 5’-CCTGGCCAGAATGCAATAACGGGAGGCGC TGTGGCTGATTTCGATAACCTGTTCGATGCTGCCATTGC CCGCGCCGATGAAACGATACGCGGGTACATGGGAACG TCAGCCACCATTACATCCGGTGAGCAGTCAGGTGCGG TGATACGTGGTGTTTTTGATGACCCTGAAAATATCAGCT ATGCCGGACAGGGCGTGCGCGTTGAAGGCTCCAGCCC GTCCCTGTTTGTCCGGACTGATGAGGTGCGGCAGCTGC GGCGTGGAGACACGCTGACCATCGGTGAGGAAAATTT CTGGGTAGATCGGGTTTCGCCGGATGATGGCGGAAGT TGTCATCTCTGGCTTGGAC-3’	IDT	N/A
lambda 200 - 5’-AAGGCGGAGAGTCAGTTCGCGGNNNN NNNNNNNNCGGCGCAACGTCGCCAGCTGTCTGCACA GGAGAAATCCCTGCTGGCGCATAAAGATGAGACGCTG GAGTACAAACGCCAGCTGGCTGCACTTGGCGACAAGG TTACGTATCAGGAGCGCCTGAACGCGCTGGCGCAGCA GGCGGATAAATTCGCACAGCAGCAA-3’	IDT	N/A
lambda 150 - 5’-GCGTCCACTGCATGTTATGCCGCGTTC GCCAGGCTTGCTGTACCATGTGCGCTGATTCTTGCGCT CAATACGTTGCAGGTTGCTTTCAATCTGTTTGTGGTATT CAGCCAGCACTGTAAGGTCTATCGGATTTAGTGCNNN NNNNNNNNN-3’	IDT	N/A
lambda 100 - 5’-TCGTTAGTTTCTCCGGTGGCAGGACGT CAGCATATTTGCTCTGGCTAATGGAGCAAAAGCGACGG GCAGGTAAAGACGTGCATTACGTNNNNNNNNNNNN-3’	IDT	N/A
lambda 75 - 5’-TCGTATCGCATTTATTGACCCGGCAAACG GGAATGAAACGCCGATGTTTGTGGCGCAGGGCAANN NNNNNNNNNN-3’	IDT	N/A
lambda 50 - 5’-ACCGCTTCCCGGTGCCGTTCACTTCCCG AATAACCCGGANNNNNNNNNNNN-3’	IDT	N/A
lambda 30 - 5’-ACGCGGTGACGACTATCAGGAAANNNNNNN-3’	IDT	N/A
I7 Extension Primer Sequence (i7 ext) - 5′-CAAGCAGAAGA CGGCATACGAGATNNNNNNNNNXXXXXXXXGTGACTG GAGTTCAGACGTGTGCTCTTCCGATCT-3’	IDT	N/A
Forward Index Primer Sequence (i5) - 5′-AATGATACGGCG ACCACCGAGATCTACACXXXXXXXXACACTCTTTCCCTA CACGACGCTCTTCCGATCT-3’	IDT	N/A
Reverse Index Primer Sequence (Ui7) - 5′- CAAGCAGAAGA CGGCATACGA-3’	IDT	N/A

**Software and algorithms**		

SRSLYumi (0.4 version)	Claret Bioscience	https://www.claretbio.com/products/software
fastp (0.23.1 version)	(Chen et al., 2018)	https://github.com/OpenGene/fastp
BWA-mem (0.7.17)	(Li and Durbin, 2009)	https://github.com/lh3/bwa
Samtools (1.9 version)	(Li et al., 2009)	http://www.htslib.org/doc/1.9/samtools.html
Qualimap (2.2.2c version)	(García-Alcalde et al., 2012)	http://qualimap.bioinfo.cipf.es/
umi-tools (11.2 version)	(Smith et al., 2017)	https://umi-tools.readthedocs.io/en/latest/index.html
Picard (2.27.0 version)	Broad Institute	http://broadinstitute.github.io/picard/
bedGraphToBigWig (4.0 version)	(Kent et al., 2010)	https://www.encodeproject.org/software/bedgraphtobigwig/
DeepTools (3.3.1 version)	(Ramírez et al., 2016)	https://deeptools.readthedocs.io/en/develop/index.html
macs2 (2.2.7.1 version)	(Zhang et al., 2008)	https://pypi.org/project/MACS2/
HOMERannotatePeaks (4.11.1 version)	(Heinz et al., 2010)	http://homer.ucsd.edu/homer/ngs/quantification.html

**Other**		

Focus-Ion Beam/Scanning Electron Microscope	FEI	Nova 200 NanoLab
Magnetic Rack 1.5mL and 15mL	Permagen	MSR6X15

### Resource availability

#### Lead contact

Further information and requests for resources and reagents should be directed to and will be fulfilled by the lead contact, David Wong (dtww@ucla.edu).

#### Materials availability

This study did not generate new unique reagents.

### Experimental model and subject details

#### Clinical samples

Plasma from healthy donors was commercially purchased from Innovative Research (IPLASK2E10ML). One donor provided whole blood collected into three vacutainers, K2EDTA, StreckDNA, and StreckRNA (Streck, 218961 and 230460). According to vendor instructions, whole blood was spun at 5000xG for 15 minutes and plasma was removed using a plasma extractor. Age and gender of the donors can be found in the supplemental chart (Table S1). Purchased samples were anonymous and did not contain any additional personal details aside from age, sex, and race and thus UCLA IRB approval was not applicable.

### Method details

#### Nucleic acid extraction

1 mL of plasma was extracted with three different methods. Using the QIAmp Circulating Nucleic Acid Kit (Qiagen, 55114) we followed two of the manufacturer protocol: Purification of Circulating Nucleic Acids from 1mL of Plasma (QiaC) and Purification of Circulating microRNA from 1mL of Plasma (QiaM). Proteinase-K digestion was carried out as instructed. Carrier RNA was not used. The ATL Lysis buffer (Qiagen, 19076) was used as indicated in the microRNA protocol. The final elution volume was 40μL.

In the magnetic bead-based uscfDNA extraction, 100μL of Proteinase K (20 mg/mL, Zymogen, D3001-2-1215) and 56μL 20% SDS (Invitrogen, AM9820) was added to 1mL of human plasma and incubated for 30minutes at 60°C. After cooling to ambient room temperature, 540μL SPRI-select beads (Beckman Coulter, B22318) and 3000μL of 100% isopropanol (Fisher, BP26181) were added to the plasma and incubated for 10 minutes on the benchtop. The plasma was then centrifuged at 4000xG for five minutes. The supernatant was removed and discarded. The pellet was resuspended using 1mL of 1x TE Buffer (Invitrogen, AM9848) and divided into 500μL aliquots into two phase lock tubes (Quantabio, 10847-802). An equal volume (500μL) of phenol:chloroform:isoamyl alcohol with equilibrium buffer was added (Sigma, P2069-100mL) and contents were vortexed for 15 seconds. The tubes were then centrifuged at 19000xG for five minutes. This was repeated twice (vortexed and centrifuged). The upper clear supernatant was pipetted and transferred to a 15mL conical tube SPRI-select beads and 3000μL of 100% isopropanol were added to the plasma and incubated for 10 minutes on the benchtop. The tube was placed on a magnetic rack for five minutes to allow for the beads to migrate. The supernatant was discarded and the beads were washed twice with 5mL of 85% ethanol. Once the second ethanol wash was removed the beads were left to air dry for 10minutes. The beads were then resuspended in 30μL of elution buffer (Qiagen, 19086) and incubated for 2 minutes. After the beads were transferred to a 1.5mL tube and magnet rack to separate the beads. Once the solution was clear (∼2 minutes) the 30μL of elution was transferred to another 1.5mL tube and combined with 1μL of 20 mg/mL glycogen (Thermo, R0561), 44μL of 1xTE Buffer, 25μL of 3M sodium acetate (Quality Biological INC, 50-751-7660), 250μL of 100% ethanol and placed at −80°C overnight. The tube was then centrifuged at 19000xG for 15 minutes. The supernatant was removed and replaced with 200μL of 80% ethanol. This was done 2 more times. The supernatant was removed and the pellet was resuspended in a 30μL of elution buffer and combined with 90μL of SPRI-select beads, 90μL of 100% isopropanol and incubated for 10 minutes. The tube was placed on a magnetic rack for five minutes to allow for the beads to migrate. The supernatant was discarded and the beads were washed twice with 200μL of 80% ethanol. Once the second ethanol wash was removed the beads were left to air dry for 10minutes. The beads were then resuspended in 40μL of Qiagen elution buffer.

#### Library preparations

Single-stranded DNA library preparation was performed using the SRSLY^TM^ PicoPlus DNA NGS Library Preparation Base Kit with the SRSLY 12 UMI-UDI Primer Set, UMI Add-on Reagents, and purified with Clarefy Purification Beads (Claret Bioscience, CBS-K250B-24, CBS-UM-24, CBS-UR-24, CBS-BD-24). Since there is currently no optimized method to measure uscfDNA, 18μL of extracted cfDNA was used as input and heat-shocked as instructed. To retain a high proportion of small fragments the low molecular weight retention protocol was followed for all bead-clean up steps. The index reaction PCR was run for 11 cycles. For double-stranded DNA libraries the NEB Ultra II (New England Bio, E7645S) was used with an 9μL aliquot of extracted cfDNA according to the manufacturer’s instructions with some modifications: the adapter ligation was performed using 2.5 μL of NEBNext® Multiplex Oligos for Illumina (Unique Dual Index UMI Adaptors RNA Set 1 - NEB, cat# E7416S); the post-adapter ligation purification was performed using 50 μL of purification beads and 50 μL of purification beads’ buffer, while the second (or post-PCR) purification was performed using 60 μL of purification beads (to retain smaller fragments). The PCR was performed using the MyTaq HS mix (Bioline, BIO-25045) for 10 PCR cycles.

#### Sequencing

Final library concentrations were measured using the Qubit Fluorometer (Thermo, Q33327) and quality assessed using the Tapestation 4200 using D1000 High-Sensitivity Tapes (Agilent, G2991BA and 5067–5584). Final libraries were sequenced on Illumina Novaseq 6000 instrument SP 300 flow cell type (2 × 150bp) at the UCLA Technology Center for Genomics & Bioinformatics.

#### Bioinformatic processing

Sequence reads were demultiplexed using SRSLYumi (SRSLYumi 0.4 version, Claret Bioscience), python package. Fastq files were trimmed with (fastp, using adapter sequence AGATCGGAAGAGCACACGTCTGAACTCCAGTCA (r1) and AGATCGGAAGAGCGTCGTGTAGGGAAAGAGTGT (r2) and a Phred score of >15. Then sequenced reads were aligned against the combined human reference genome [GenBank:GCA_000001305.2] and LambdaPhage Genome [GeneBank:GCA_000840245.1] using BWA-mem. http://broadinstitute.github.io/picard/). Samples were sorted and filtered using samtools (1.9 version). Reads were deduplicated by first moving the umi-tag using the bamtag tool from SRSLYumi (0.4 version), grouping with umi-tools (11.2 version), and <!--Q1: According to which tool? Picard or it is based on UMI information?-- > removed using markduplicates from the Picard Toolkit (Quality control was performed with Qualimap (2.2.2c version). UMI-duplicate removal was done first by moving the UMI-tag with srslyumi-bamtag(SRSLYumi), marking with umi-tools (11.2 version), then removal with Picard (2.27.0 version). Bam files were split by size (uscfDNA 25–100 and mncfDNA 101–250) using alignmentSieve in deepTools (3.31 version). Correlation heatmaps were generated using bedGraphToBigWig (version 4.0) and plotCorrelation in DeepTools (3.31 version). Functional peaks were first called with macs2 (2.2.7.1 version) and then analyzed with HOMERannotatePeaks (version 4.11.1).

#### Nuclease digestions for analysis of strandedness

Prior to library preparation, the extracted cfDNA was digested with various strand-specific nucleases. For all reactions 500pg of control oligos (350nt ssDNA and 460bp dsDNA lambda sequence, IDT) was spiked into 20μL of extracted cfDNA. After the reaction, the DNA was purified by combining 30μL of reaction buffer and 90μL of SPRI-select beads, 90μL of 100% isopropanol and incubated for 10 minutes. The tube was placed on a magnetic rack for five minutes to allow for the beads to migrate. The supernatant was discarded and the beads were washed twice with 200μL of 80% ethanol. Once the second ethanol wash was removed the beads were left to air dry for 10 minutes. The beads were then resuspended in 20μL of Qiagen elution buffer (or TrisHCl pH 8 10 mM).

##### Non-strand specific DNA digestion

20μL cfDNA was combined with 1μL **DNase I** (Invitrogen, 18-068-015), 3μL 10xDNase 1 Buffer, 6μL of ddH_2_O incubated for 15minutes at 37°C and heat inactivated for 15 minutes at 80°C with 1μL of 0.5M EDTA.

##### ssDNA-specific digestion

20μL cfDNA was combined with 1μL 1x **S1** (Thermo, EN0321), 6μL 5x S1 Buffer, 3μL of ddH_2_O incubated for 30 minutes at room temperature and heat inactivated for 15 minutes at 80°C with 2μL of 0.5M EDTA.

##### ssDNA-specific digestion

20μL cfDNA was combined with 1μL 0.1x **P1** (NEB, M0660S), 3μL NEBuffer r1.1, 6μL of ddH_2_O incubated for 30 minutes at 37°C and inactivated with 2μL of 0.5M EDTA.

##### ssDNA-specific digestion

20μL cfDNA was combined with 3μL **Exonuclease 1** (NEB, M0293S), 3μL 10x Exo 1 Buffer, 4μL of ddH_2_O incubated for 30 minutes at 37°C and heat inactivated for 15 minutes at 80°C with 1μL of 0.5M EDTA.

##### dsDNA-specific digestion

20μL cfDNA was combined with 2μL **dsDNase** (ArcticZyme, 70600-201), 8μL of ddH_2_O incubated for 30 minutes at 37°C and heat inactivated for 15 minutes at 65°C with 1mM DTT.

##### Nick repair analysis

20μL cfDNA was combined with 1μL **PrePCR Repair** (NEB, M0309S), 5μL ThermoPol Buffer (10x), 0.5μL of NAD+ (100x), 2μL of Takara 2.5mM dNTP, 21.5 ddH_2_O incubated for 30 minutes at 37°C and placed on ice.

##### RNA digestion

20μL of cfDNA was combined with 1μL of **RNase Cocktail** (Thermo, AM228). For 20 minutes at 30°C prior to input into the library preparation.

#### ssDNA ladder to determine efficiency

2ng ssDNA ladder of various sizes (30–200) was spiked in 1mL healthy plasma prior to extraction. Final elution was 40μL and 18μL was used for each final library. Oligonucleotides were manufactured by a commercial vendor (IDT, Custom Order).

#### Scanning electron microscope (SEM)

After processing PBS or plasma samples with QiaC or QiaM protocol, the columns were air-dried at room temperature. They were cut into proper height to expose the membrane and fitted to the sample stage. The samples were coated with platinum and the detailed morphology of the membrane was examined by Focus-Ion Beam/Scanning Electron Microscopy (FEI, Nova 200 NanoLab).

### Quantification and statistical analysis

The quantification of “%uscfDNA” in Figure 2B was performed by calculating the ratio of the sample intensity (FU) of the electropherogram images between the ultrashort region (180-250bp) and the mncfDNA (251-350bp). Similarly, sample intensity was used to calculate the fold change of %Area cfDNA to control (Figure 3B). A paired two-tailed student-test test was performed after ANOVA analysis in order to determine statistical significance. ∗ *p* *< 0.05, ∗∗* *p* *< 0.01, and ∗∗∗* *p* *< 0.001*. Bars graphs represent standard error of Mean (SEM).

## Data Availability

• All original code is available from the lead contact upon request. • The accession number for the raw .fastq files of the plasma samples and peaks calls reported in this paper is NCBI GEO: GSE202433. • Any additional information required to reanalyze the data reported in this paper is available from the lead contact upon request.
