# Supplementary material for: Plasma contains ultrashort single-stranded DNA in addition to nucleosomal cell-free DNA
Source: iScience. 2022 Jun 8;25(7):104554. doi: 10.1016/j.isci.2022.104554 (PMC9254344; doi:10.1016/j.isci.2022.104554)
Supplement: Document S1. Figures S1–S8 and Tables S1–S9 [file mmc1.pdf]

iScience, Volume *25*

Supplemental information

Plasma contains ultrashort single-stranded DNA in addition to nucleosomal cell-free DNA

Jordan Cheng, Marco Morselli, Wei-Lun Huang, You Jeong Heo, Thalyta Pinheiro-Ferreira, Feng Li, Fang Wei, David Chia, Yong Kim, Hua-Jun He, Kenneth D. Cole, Wu-Chou Su, Matteo Pellegrini, and David T.W. Wong

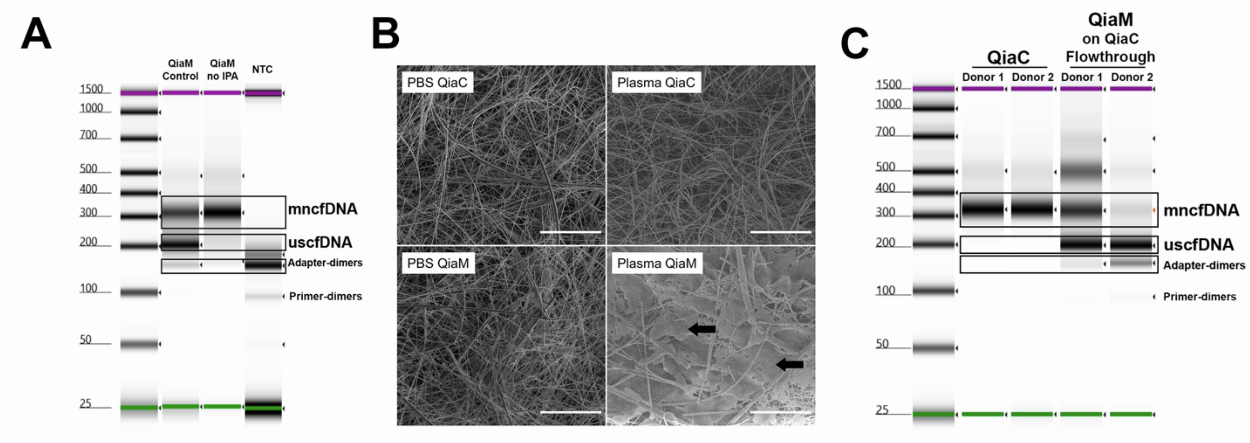

Figure S1. Inherent characteristics of the QiaM extraction protocol (related to Figure 2). (**A)** The increased isopropanol (1.8ml to 2.3ml) is integral to retaining the uscfDNA from plasma. (**B)** SEM images of the Qiagen silica filter show global sheet-like deposits (black arrows) only in QiaM extraction of plasma. Scale bars (white line) represents 50uM. **(C)** Using the Qiagen Kit with a centrifuge (as opposed to vacuum), the flow through from a QiaC plasma extraction was subsequently extracted with QiaM to reveal the rescue of the uscfDNA band.

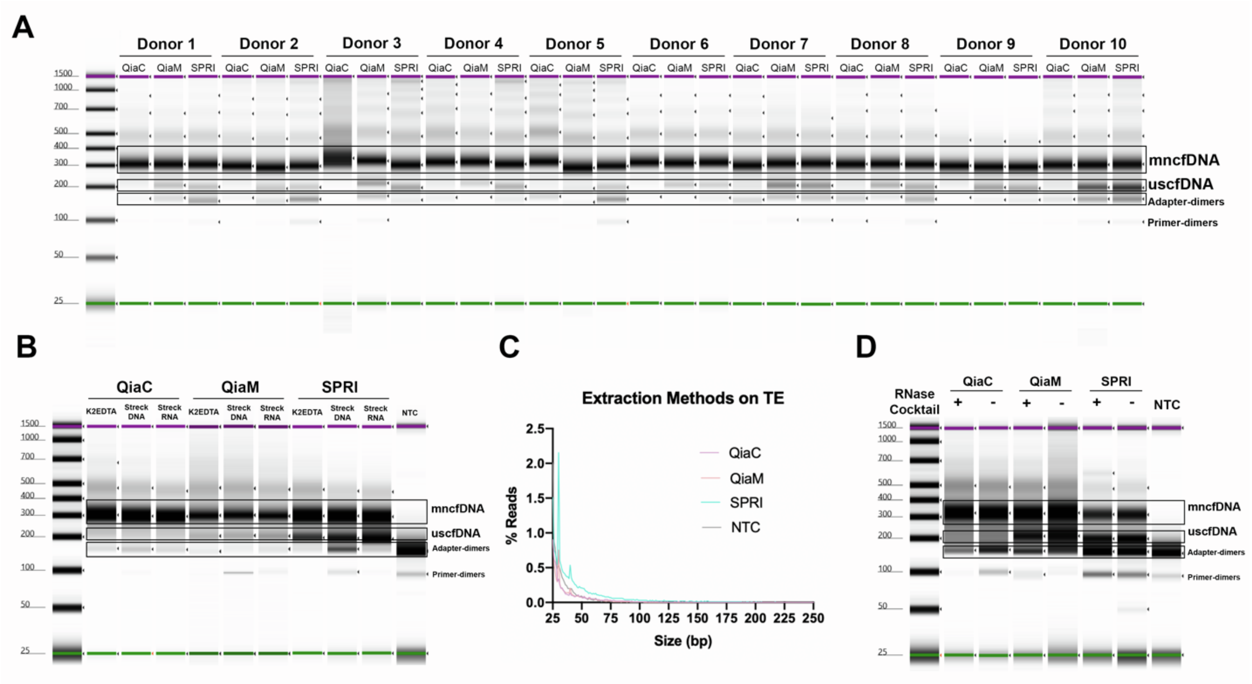

Figure S2. UscfDNA can be consistently observed (related to Figure 2B). (A) Electropherogram images of ten healthy donors extracted with QiaC, QiaM, and SPRI showing the presence of uscfDNA. (**B)** UscfDNA exists independently of the whole blood collection tube. (**C)** TE buffer control extracted with the three methods do not produce uscfDNA or mncfDNA peaks when aligned to the human genome. **(D)** RNase cocktail digestion prior to library preparation does not reduce the uscfDNA band in QiaM and SPRI extracted samples.

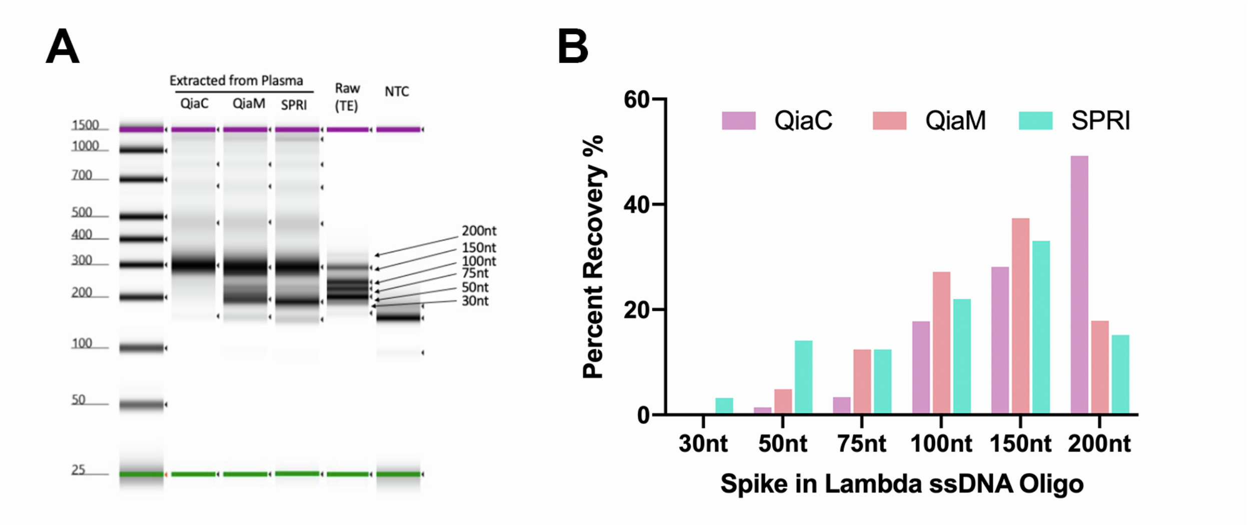

Figure S3. Magnetic bead extraction methods may capture short and single-stranded DNA molecules better than silica column-based methods (related to Figure 2). (A) Extraction of healthy plasma spiked with a ladder of short lambda ssDNA oligos shows various retention efficiencies between QiaC, QiaM and SPRI methods. **(B)** After alignment to the lambda genome shows QiaM and SPRI methods have greater efficiency of extracting ultrashort ssDNA molecules.

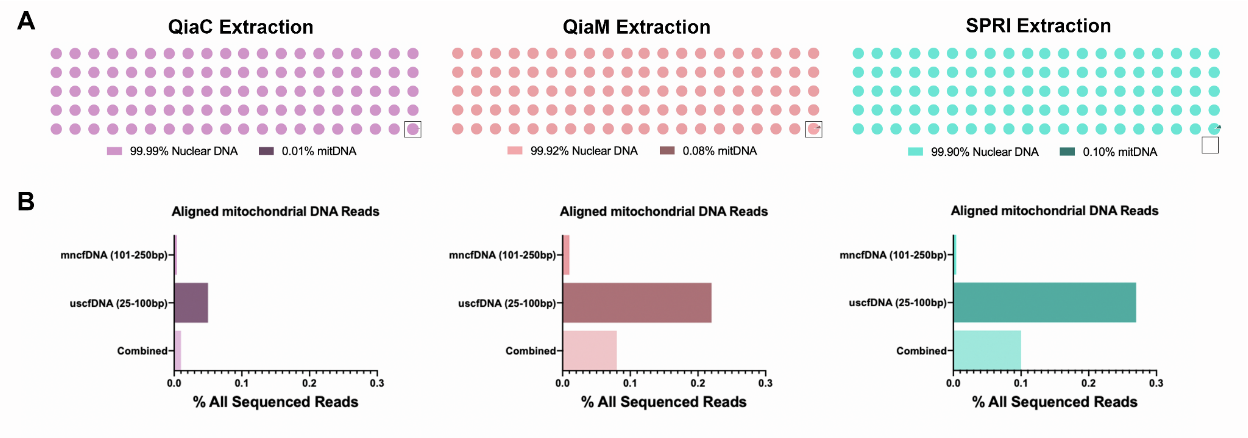

Figure S4. Mitochondrial contribution to cfDNA (related to Figure 2). (**A)** The majority of DNA aligns to the nuclear genome and not to the mitochondrial genome. Square indicates the visual representation of contribution of mitochondria reads. Extraction methods: QiaC (fuchsia), QiaM (pink), and SPRI (teal). (**B)** QiaM and SPRI are enriched for mitochondrial DNA in the uscfDNA population but still is a minor fraction of total DNA.

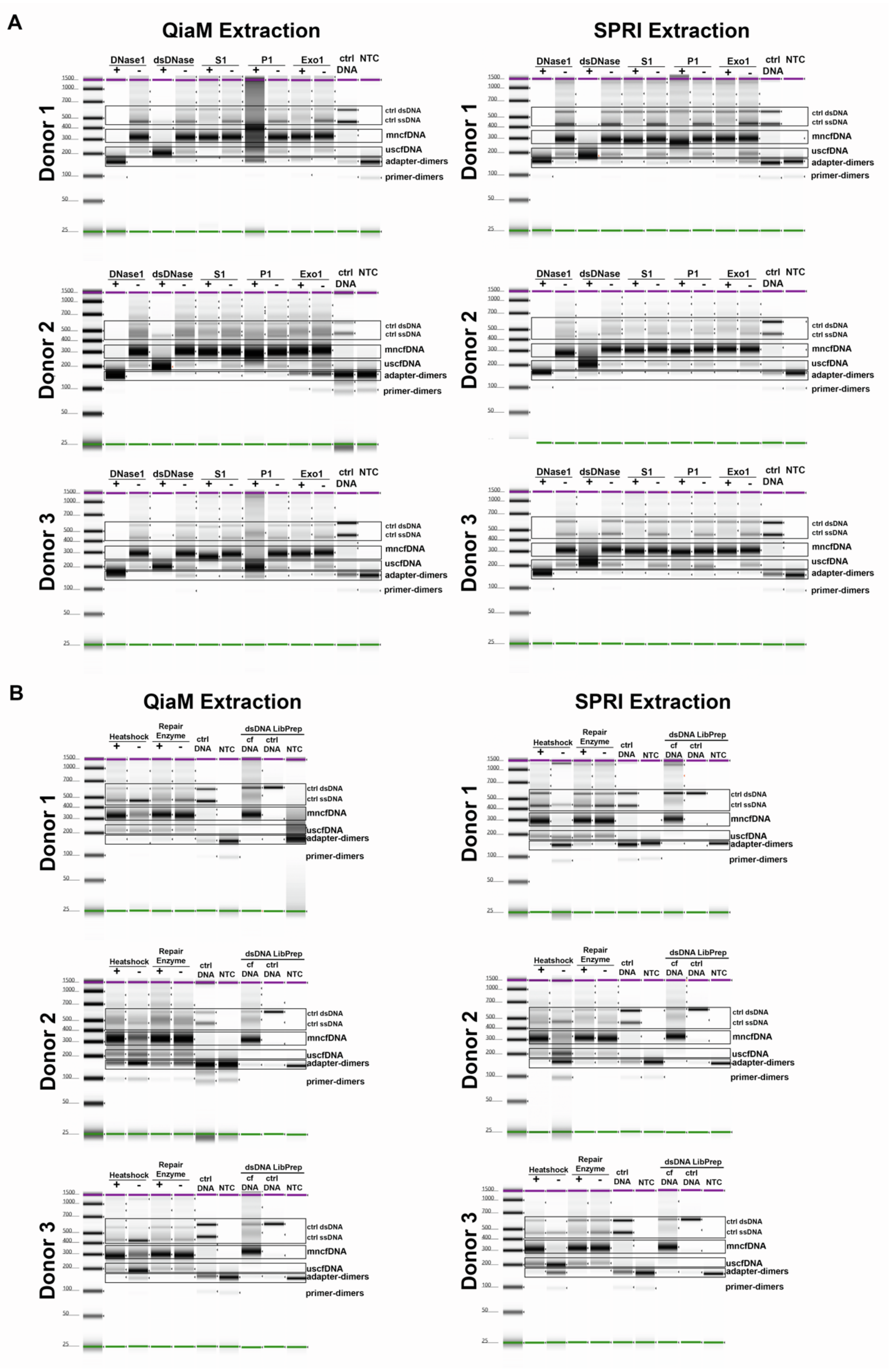
A
QiaM Extraction
SPRI Extraction
DNase1
dsDNase
S1
P1
Exo1
ctrl DNA
NTC
+
-
Donor 1
Donor 2
Donor 3
1500
1000
700
500
400
300
200
100
50
25
ctrl dsDNA
ctrl ssDNA
mncfDNA
uscfDNA
adapter-dimers
primer-dimers
B
QiaM Extraction
SPRI Extraction
Heatshock
Repair Enzyme
ctrl DNA
NTC
dsDNA LibPrep
cf DNA
ctrl DNA
NTC

Figure S5. Electropherograms of final libraries prepared from different treatments (related to Figure 3). (A) Electropherograms of final libraries constructed from extracted cfDNA after nuclease digestion. (**B)** Electropherograms of final libraries constructed from extracted cfDNA after undergoing ssDNA, dsDNase library preparation and nick-repair enzyme treatment. Replicate experiments using plasma from three healthy donors extracted by QiaM and SPRI.

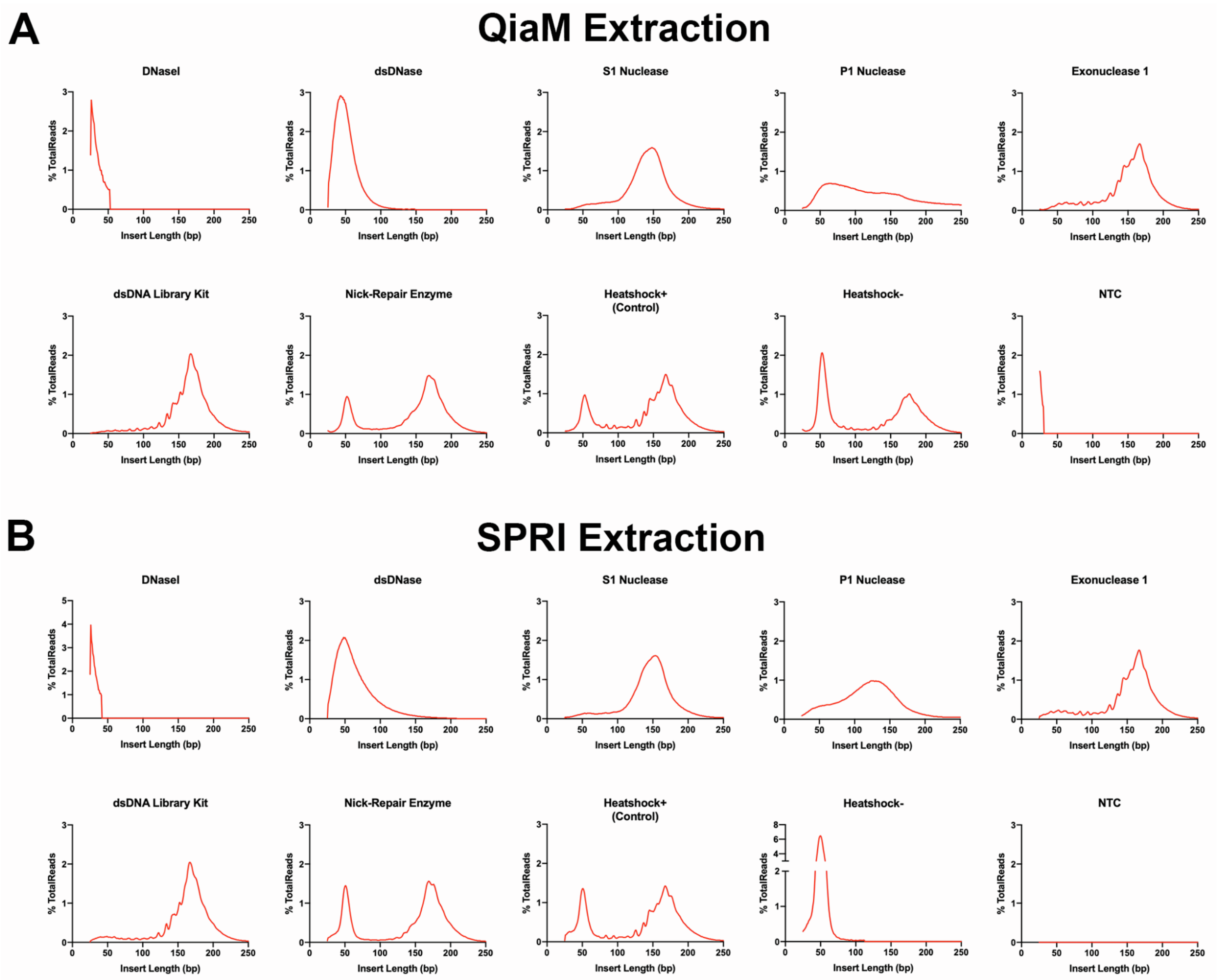

Figure S6. Fragment length distribution of aligned reads from samples that underwent digestions or variations in the library prep method (related to Figure 3). Alignment of sequenced libraries to human genome pretreated by digestions and library preparation variations from Donor 1 of Sup Fig 3 extracted by QiaM (**A**) and SPRI (**B**). Reads with insert size under 25bp and above 250bp were excluded from the plots.

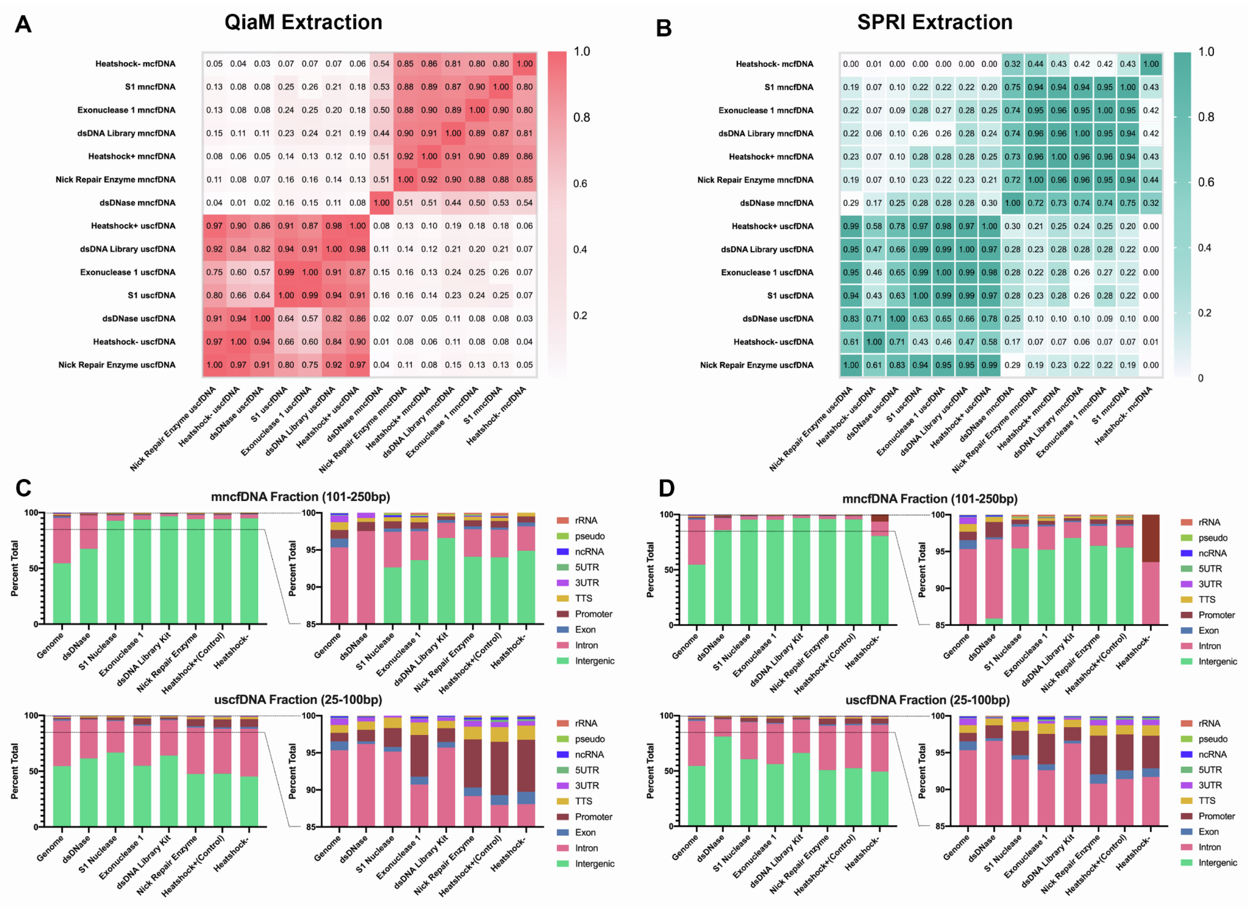

Figure S7. Heatmap correlation uscfDNA and mncfDNA reads (related to Figure 3). Heatmap correlation uscfDNA and mncfDNA reads of various digestions of samples extracted by QiaM (**A**) and SPRI (**B**). Individual functional element peak analysis of sequenced reads from digestions of QiaM (**C**) and SPRI (**D**) from Figure 3. Values are summated in Figure 4.

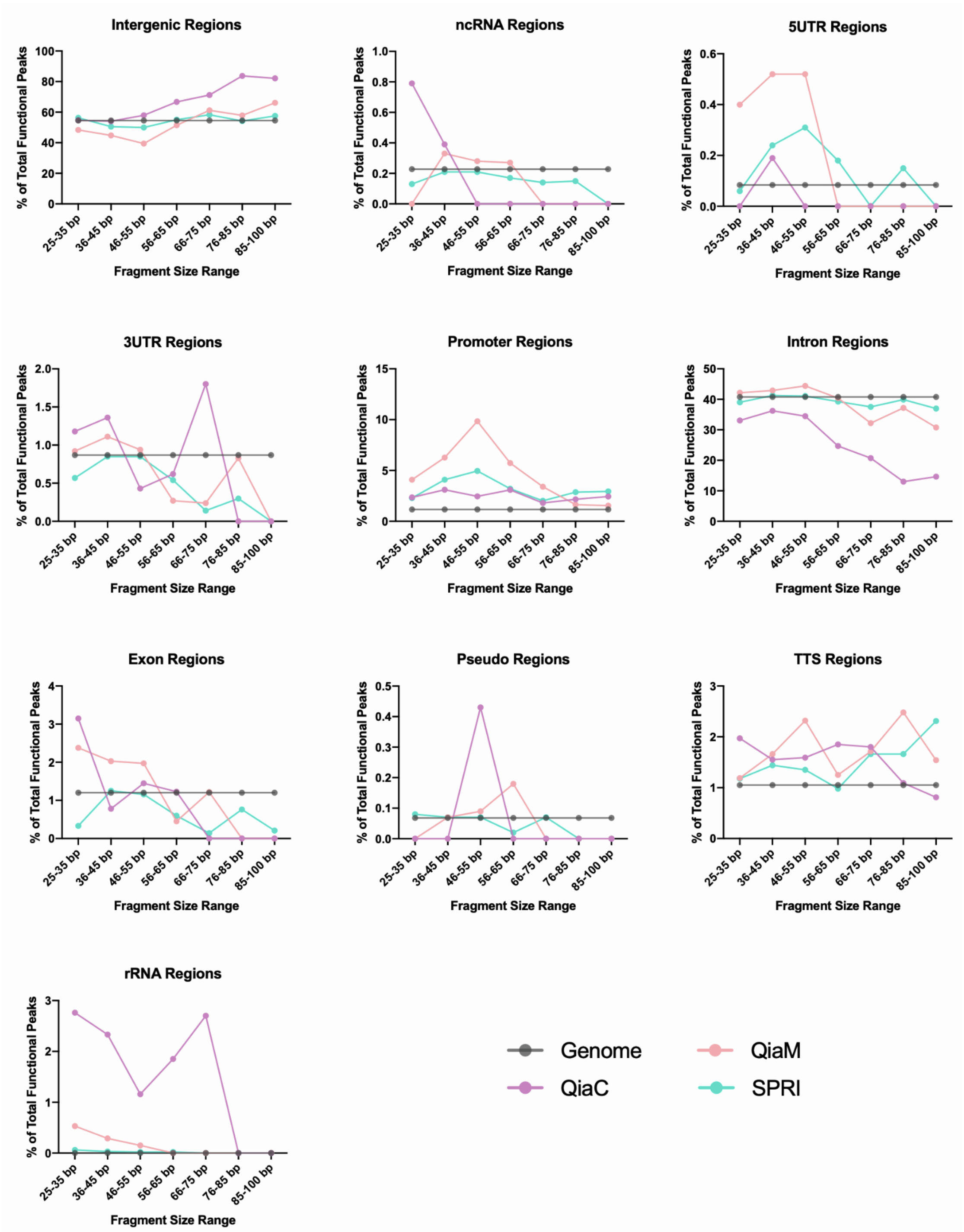

Figure S8. The proportion of functional peaks at different fragment sizes (related to Figure 4). Functional peaks were first called with macs2 (2.2.7.1 version) and then analyzed with HOMERannotatePeaks (version 4.11.1).

Table S1. Plasma Donor Information (related to STAR Methods)

Assay	Gender	Age
Digestions Donor 1	Male	47
Digestions Donor 2	Female	57
Digestions Donor 3	Male	35
Healthy 10 Replicate Donor 1	Male	45
Healthy 10 Replicate Donor 2	Male	18
Healthy 10 Replicate Donor 3	Male	23
Healthy 10 Replicate Donor 4	Male	26
Healthy 10 Replicate Donor 5	Male	38
Healthy 10 Replicate Donor 6	Male	33
Healthy 10 Replicate Donor 7	Male	22
Healthy 10 Replicate Donor 8	Male	37
Healthy 10 Replicate Donor 9	Male	27
Healthy 10 Replicate Donor 10	Male	41
Healthy Donor for QiaM on QiaC Flowthrough 1	Male	19
Healthy Donor for QiaM on QiaC Flowthrough 2	Male	25

Table S2. Synthetic Oligomers and Primers (related to STAR Methods)

<table>
<tr><th>Name</th><th>Size</th><th>ss/ds</th><th>Lambda phage region</th><th>Notes</th></tr>
<tr><td>Lambda dsDNA Control</td><td>459 bp</td><td>ds</td><td>27’944:28’402</td><td>PCR product, no UMI</td></tr>
<tr><td colspan="5">5’-
CAAACTGCGCAACTCGTGAAAGGTAGGCGGATCCCCTTCGAAGGAAAGACCTGATGCTTTTCGTG
CGCGCATAAAATACCTTGATACTGTGCCGGATGAAAGCGGTTCGCGACGAGTAGATGCAATTATG
GTTTCTCCGCCAAGAATCTCTTTGCATTTATCAAGTGTTTCCTTCATTGATATTCCGAGAGCATCAAT
ATGCAATGCTGTTGGGATGGCAATTTTTACGCCTGTTTTGCTTTGCTCGACATAAAGATATCCATCT
ACGATATCAGACCACTTCATTTCGCATAAATCACCAACTCGTTGCCCGGTAACAACAGCCAGTTCC
ATTGCAAGTCTGAGCCAACATGGTGATGATTCTGCTGCTTGATAAATTTTCAGGTATTCGTCAGCC
GTAAGTCTTGATCTCCTTACCTCTGATTTTGCTGCGCGAGTGGCAGCGACATGGTTTGTTGT-3’</td></tr>
<tr><td>Lambda ssDNA Control</td><td>350 nt</td><td>ss</td><td>7’582:7’930</td><td>IDT synthesized</td></tr>
<tr><td colspan="5">5’-
CCTGGCCAGAATGCAATAACGGGAGGCGCTGTGGCTGATTTCGATAACCTGTTCGATGCTGCCAT
TGCCCGCGCCGATGAAACGATACGCGGGTACATGGGAACGTCAGCCACCATTACATCCGGTGAG
CAGTCAGGTGCGGTGATACGTGGTGTTTTTGATGACCCTGAAAATATCAGCTATGCCGGACAGGG
CGTGCGCGTTGAAGGCTCCAGCCCGTCCCTGTTTGTCCGGACTGATGAGGTGCGGCAGCTGCGG
CGTGGAGACACGCTGACCATCGGTGAGGAAAATTTCTGGGTAGATCGGGTTTCGCCGGATGATGG
CGGAAGTTGTCATCTCTGGCTTGGAC-3’</td></tr>
<tr><td>lambda 200</td><td>198 nt</td><td>ss</td><td>12’051:12’248</td><td>IDT synthesized, internal-UMI 12nt</td></tr>
<tr><td colspan="5">5’-
AAGGCGGAGAGTCAGTTCGCGGNNNNNNNNNNNNCGGCGCAACGTCGCCAGCTGTCTGCACAG
GAGAAATCCCTGCTGGCGCATAAAGATGAGACGCTGGAGTACAAACGCCAGCTGGCTGCACTTGG
CGACAAGGTTACGTATCAGGAGCGCCTGAACGCGCTGGCGCAGCAGGCGGATAAATTCGCACAG
CAGCAA-3’</td></tr>
<tr><td>lambda 150</td><td>150 nt</td><td>ss</td><td>35’073:35’201+UMI</td><td>IDT synthesized, 3’-UMI 12nt</td></tr>
<tr><td colspan="5">5’-
GCGTCCACTGCATGTTATGCCGCGTTCGCCAGGCTTGCTGTACCATGTGCGCTGATTCTTGCGCT
CAATACGTTGCAGGTTGCTTTCAATCTGTTTGTGGTATTCAGCCAGCACTGTAAGGTCTATCGGATT
TAGTGCNNNNNNNNNNNN-3’</td></tr>
<tr><td>lambda 100</td><td>100 nt</td><td>ss</td><td>41’091:41’178+UMI</td><td>IDT synthesized, 3’-UMI 12nt</td></tr>
<tr><td colspan="5">5’-
TCGTTAGTTTCTCCGGTGGCAGGACGTCAGCATATTTGCTCTGGCTAATGGAGCAAAAGCGACGG
GCAGGTAAAGACGTGCATTACGTNNNNNNNNNNNN-3’</td></tr>
<tr><td>lambda 75</td><td>75 nt</td><td>ss</td><td>18’204:18’266+UMI</td><td>IDT synthesized, 3’-UMI 12nt</td></tr>
</table>

5’-TCGTATCGCATTTATTGACCCGGCAAACGGGAATGAAACGCCGATGTTTGTGGCGCAGGGCAANNNNNNNNNNNN-3’				
lambda 50	50 nt	ss	2’321:2’359+UMI	IDT synthesized, 3’-UMI 12nt
5’-ACCGCTTCCCGGTGCCGTTCACTTCCCGAATAACCCGGANNNNNNNNNNNN-3’				
lambda 30	30 nt	ss	4’278:4’300+UMI	IDT synthesized, 3’-UMI 9nt
5’-ACGCGGTGACGACTATCAGGAAANNNNNNN-3’				
I7 Extension Primer Sequence (i7 ext)	75 nt	5′-CAAGCAGAAGACGGCATACGAGATNNNNNNNNNXXXXXXXXGTGACTGGAGTTCAGACGTGTGCTCTTCCGATCT-3’		
Forward Index Primer Sequence (i5)	70 nt	5′-AATGATACGGCGACCACCGAGATCTACACXXXXXXXXACACTCTTTCCCTACACGACGCTCTTCCGATCT-3’		
Reverse Index Primer Sequence (Ui7)	21 nt	5′- CAAGCAGAAGACGGCATACGA-3’		

Table S3. Numerical values related to Figure 2B.

uscfDNA region/total cfDNA (uscfDNA + mncfDNA)	Donor 1	Donor 2	Donor 3	Donor 4	Donor 5	Donor 6	Donor 7	Donor 8	Donor 9	Donor 10	Mean
QiaC	7.04%	7.47%	5.00%	7.93%	7.81%	4.91%	11.80%	5.82%	8.39%	10.67%	7.68%
QiaM	16.96%	17.77%	23.29%	11.44%	10.53%	13.15%	25.79%	11.63%	22.85%	32.18%	18.56%
SPRI	17.63%	15.98%	21.59%	18.70%	11.44%	12.55%	24.51%	13.64%	22.68%	37.10%	19.58%

Table S4. NGS Statistics related to Figure 2.

	Total Reads Sequenced (Million)	Reads After Adapter Removal and Quality Filtering (Million)	Reads Mapped to Human Genome and Lambda (Million)	Reads Mapped to Human Genome Only (Million)	Human Genome Only Map Percentage [A]	Mean Human Genome Coverage	uscfDNA Reads Mapping (25-100bp) (Million)	% Proportion of uscfDNA Reads [B]	mncfDNA Reads Mapping (101-250bp) (Million)	% Proportion of mncfDNA Reads [C]	Remaining Reads (<25 & > 250bp) (Million)	% Proportion of Remaining Reads [D]
QiaC	**127.4**	**119.2**	**116.4**	**110.0**	97.5%	5.05	**12.2**	11.1%	**88.0**	80.0%	**9.7**	8.86%
QiaM	**111.7**	**101.8**	**98.2**	**67.8**	94.9%	3.23	**23.2**	34.2%	**42.8**	63.1%	**1.8**	2.63%
SPRI	**135.4**	**118.0**	**116.9**	**86.5**	98.8%	3.75	**31.2**	36.1%	**53.2**	61.6%	**2.0**	2.33%

[A] Human Genome Only Map Percentage: $\frac{reads\ mapped\ to\ human\ genome}{all\ reads\ after\ QC\ and\ adapter\ removal - Lambda\ reads}$

[B] % Proportion of uscfDNA Reads: $\frac{uscfDNA\ reads\ mapped\ to\ human\ genome}{all\ reads\ mapped\ to\ human\ genome}$

[C] % Proportion of mncfDNA Reads: $\frac{mncfDNA\ reads\ mapped\ to\ human\ genome}{all\ reads\ mapped\ to\ human\ genome}$

[D] % Proportion of Remaining Reads: $\frac{remaining\ reads\ mapped\ to\ human\ genome}{all\ reads\ mapped\ to\ human\ genome}$

Table S5. Numerical values related to Figure 2F.

Annotation	Genome	QiaC mncfDNA	QiaM mncfDNA	SPRI mncfDNA	QiaC uscfDNA	QiaM uscfDNA	SPRI uscfDNA
rRNA	0.00%	0.23%	0.43%	0.15%	0.51%	0.17%	0.01%
pseudo	0.07%	0.06%	0.12%	0.11%	0.08%	0.10%	0.06%
ncRNA	0.23%	0.02%	0.12%	0.00%	0.16%	0.28%	0.18%
5UTR	0.08%	0.00%	0.00%	0.00%	0.11%	0.32%	0.26%
3UTR	0.87%	0.00%	0.06%	0.00%	0.53%	0.62%	0.73%
TTS	1.05%	0.21%	0.37%	0.33%	1.23%	1.96%	1.23%
Promoter	1.16%	0.45%	0.73%	0.41%	3.42%	8.04%	4.44%
Exon	1.20%	0.17%	0.24%	0.15%	1.12%	1.41%	1.12%
Intron	40.76%	2.44%	3.41%	2.43%	34.83%	42.55%	39.94%
Intergenic	54.57%	96.41%	94.52%	96.42%	58.01%	44.55%	52.01%

Table S6. NGS Statistics related to Figure 3A and B.

QiaM Extraction Method					
Treatment	Total Reads Sequenced (bases)	Reads After Adapter Removal and Quality Filtering (bases)	Total Reads Mapped to Human and Lambda Genome (bases)	Human and Lambda Genome Map %	Mean Coverage
DNaseI	8393732	1065752	523570	49.1%	0.005
dsDNase	115213972	111823988	109985202	98.4%	1.971
S1 Nuclease	60057996	56388884	55103368	97.7%	2.340
P1 Nuclease	89318504	70739876	68063693	96.2%	2.531
Exonuclease 1	88988296	74732014	72354884	96.8%	3.123
dsDNA Library Kit	62108730	58650304	58270173	99.4%	2.669
Nick-Repair	54468958	50417951	47386326	94.0%	1.862
Heatshock+	110037748	103970389	99647927	95.8%	4.007
Heatshock-	41838040	39416324	36961704	93.8%	1.360
NTC	2514676	230826	114609	49.7%	0.001

SPRI Extraction Method					
Treatment	Total Reads Sequenced (bases)	Reads After Adapter Removal and Quality Filtering (bases)	Total Reads Mapped to Human and Lambda Genome (bases)	Human and Lambda Genome Map %	Mean Coverage
DNaseI	8466754	1974781	1162689	58.9%	0.010
dsDNase	185175564	183938733	183099719	99.5%	3.882
S1 Nuclease	104009908	98239896	97290270	99.0%	4.155
P1 Nuclease	165942090	148324496	146379814	98.7%	5.597
Exonuclease 1	186861886	160648904	158024760	98.4%	6.891
dsDNA Library Kit	101486352	97757377	97617788	99.9%	4.463
Nick-Repair	169084738	152995334	150434158	98.3%	6.094
Heatshock+	233954594	218,543,557	218,543,557	99.2%	8.853
Heatshock-	11121808	5508994	5375857	97.6%	0.153
NTC	6194542	450739	225630	50.1%	0.002

Table S7. Numerical values related to Figure 3A and B.

		QIAM				SPRI				QIAM				SPRI			
Donor		dsDNase +	S1+	Exo1	Control	dsDNase +	S1+	Exo1	Control	Heatshock	No Heat Shock	dsDNA Library	Repair Enzyme	Heatshock	No Heat Shock	dsDNA Library	Repair Enzyme
1A	uscfDNA (%integrated area of the intensity of 180-250bp)	66.01	3.43	6.90	9.31	64.37	7.13	6.55	12.53	9.31	14.14	0.60	9.87	12.53	21.23	2.03	11.98
	mncfDNA (%integrated area of the intensity of 250-400bp)	21.38	56.79	51.50	49.2	29.23	47.70	43.49	43.44	49.20	37.39	37.20	46.57	43.44	12.37	41.98	40.41
	uscfDNA fold signal from control or heatshock	7.09	0.37	0.74	1.00	5.14	0.57	0.52	1.00	1.00	1.52	0.06	1.06	1.00	1.69	0.16	0.96
	mncfDNA fold signal from control or heatshock	0.43	1.15	1.05	1.00	0.67	1.10	1.00	1.00	1.00	0.76	0.76	0.95	1.00	0.28	0.97	0.93
2A	uscfDNA (%integrated area of the intensity of 180-250bp)	69.13	3.53	5.87	11.93	48.15	7.16	4.99	11.12	11.93	17.96	1.85	8.01	9.12	26.28	2.88	8.01
	mncfDNA (%integrated area of the intensity of 250-400bp)	18.02	68.28	56.93	52.58	36.94	64.80	61.32	57.75	52.58	32.03	51.89	54.18	61.02	23.21	51.84	54.18
	uscfDNA fold signal from control or heatshock	5.79	0.30	0.49	1.00	4.33	0.64	0.45	1.00	1.00	1.51	0.16	0.67	1.00	2.88	0.32	0.88
	mncfDNA fold signal from control or heatshock	0.34	1.30	1.08	1.00	0.64	1.12	1.06	1.00	1.00	0.61	0.99	1.03	1.00	0.38	0.85	0.89
3A	uscfDNA (%integrated area of the intensity of 180-250bp)	74.19	8.78	6.98	13.62	41.43	7.94	3.47	18.92	13.62	30.92	1.73	12.96	13.62	30.92	3.82	12.96
	mncfDNA (%integrated area of the intensity of 250-400bp)	14.43	61.55	59.99	54.73	45.39	55.38	57.62	52.9	54.73	31.80	45.66	50.99	54.73	31.80	53.96	50.99
	uscfDNA fold signal from control or heatshock	5.45	0.64	0.51	1.00	2.19	0.42	0.18	1.00	1.00	2.27	0.13	0.95	1.00	2.27	0.28	0.95
	mncfDNA fold signal from control or heatshock	0.26	1.12	1.10	1.00	0.86	1.05	1.09	1.00	1.00	0.58	0.83	0.93	1.00	0.58	0.99	0.93
Mean	uscfDNA fold signal from control	6.11	0.44	0.58	1	3.89	0.54	0.38	1	1.00	1.76	0.12	0.89	1.00	2.28	0.25	0.93
	mncfDNA fold signal from control	0.35	1.19	1.08	1	0.72	1.09	1.05	1	1.00	1.76	0.12	0.89	1.00	2.28	0.25	0.93

Table S8. Numerical values related to Figure 4A and B and Supplemental Figure 5C and D.

	QiaM mncfDNA										
Annotation	Genome	DNaseI mncfDNA	dsDNase mncfDNA	S1 Nuclease mncfDNA	P1 Nuclease mncfDNA	Exonuclease 1 mncfDNA	dsDNA Library Kit mncfDNA	Nick Repair Enzyme mncfDNA	Heatshock+(Control) mncfDNA	Heatshock- mncfDNA	NTC mncfDNA
Intergenic	54.573%	92.37%	67.54%	92.63%	87.72%	93.63%	96.60%	94.08%	94.01%	94.88%	0%
Intron	40.756%	4.79%	30.00%	4.81%	5.55%	3.88%	2.05%	3.70%	3.71%	3.30%	0%
Exon	1.203%	0.44%	0.00%	0.44%	2.04%	0.33%	0.37%	0.40%	0.29%	0.50%	0%
Promoter	1.165%	0.98%	1.23%	0.98%	3.06%	0.88%	0.49%	0.81%	0.88%	0.83%	0%
TTS	1.050%	0.54%	0.53%	0.55%	0.53%	0.66%	0.25%	0.47%	0.44%	0.50%	0%
3UTR	0.869%	0.00%	0.70%	0.00%	0.15%	0.00%	0.00%	0.07%	0.00%	0.00%	0%
5UTR	0.084%	0.00%	0.00%	0.00%	0.49%	0.00%	0.00%	0.00%	0.00%	0.00%	0%
ncRNA	0.228%	0.27%	0.00%	0.27%	0.23%	0.18%	0.00%	0.07%	0.18%	0.00%	0%
pseudo	0.068%	0.27%	0.00%	0.27%	0.15%	0.18%	0.08%	0.20%	0.11%	0.00%	0%
rRNA	0.001%	0.33%	0.00%	0.33%	0.08%	0.26%	0.16%	0.20%	0.37%	0.00%	0%

	QiaM uscfDNA										
Annotation	Genome	DNaseI uscfDNA	dsDNase uscfDNA	S1 Nuclease uscfDNA	P1 Nuclease uscfDNA	Exonuclease 1 uscfDNA	dsDNA Library Kit uscfDNA	Nick Repair Enzyme uscfDNA	Heatshock+(Control) uscfDNA	Heatshock- uscfDNA	NTC uscfDNA
Intergenic	54.573%	56.80%	61.44%	66.75%	56.43%	54.92%	64.02%	47.54%	47.77%	45.24%	60.33%
Intron	40.756%	36.40%	34.76%	28.41%	27.03%	35.80%	31.69%	41.61%	40.20%	42.84%	30.58%
Exon	1.203%	0.84%	0.35%	0.62%	8.42%	1.06%	0.74%	1.16%	1.32%	1.65%	3.31%
Promoter	1.165%	2.93%	1.54%	2.55%	4.45%	5.61%	1.86%	6.49%	7.18%	7.03%	3.31%
TTS	1.050%	1.26%	1.13%	1.41%	1.35%	1.69%	1.00%	1.72%	1.99%	1.90%	0.00%
3UTR	0.869%	0.94%	0.51%	0.26%	0.74%	0.48%	0.46%	0.79%	0.69%	0.62%	0.83%
5UTR	0.084%	0.00%	0.03%	0.00%	0.25%	0.06%	0.06%	0.14%	0.29%	0.26%	0.00%
ncRNA	0.228%	0.10%	0.14%	0.18%	0.52%	0.24%	0.17%	0.29%	0.34%	0.28%	0.00%
pseudo	0.068%	0.00%	0.06%	0.18%	0.65%	0.09%	0.00%	0.14%	0.16%	0.08%	0.00%
rRNA	0.001%	0.73%	0.04%	0.09%	0.15%	0.06%	0.00%	0.14%	0.05%	0.11%	1.65%

	SPRI mncfDNA										
Annotation	Genome	DNaseI mncfDNA	dsDNase mncfDNA	S1 Nuclease mncfDNA	P1 Nuclease mncfDNA	Exonuclease 1 mncfDNA	dsDNA Library Kit mncfDNA	Nick Repair Enzyme mncfDNA	Heatshock+(Control) mncfDNA	Heatshock- mncfDNA	NTC mncfDNA
Intergenic	54.573%	37.50%	85.90%	95.40%	94.86%	95.24%	96.82%	95.77%	95.53%	80.65%	0%
Intron	40.756%	50.00%	10.75%	2.98%	3.24%	3.18%	2.17%	2.70%	2.97%	12.90%	0%
Exon	1.203%	0.00%	0.27%	0.34%	0.27%	0.22%	0.18%	0.28%	0.19%	0.00%	0%
Promoter	1.165%	0.00%	2.08%	0.61%	0.78%	0.52%	0.36%	0.61%	0.61%	6.45%	0%
TTS	1.050%	0.00%	0.67%	0.24%	0.41%	0.37%	0.26%	0.28%	0.32%	0.00%	0%
3UTR	0.869%	0.00%	0.00%	0.00%	0.02%	0.00%	0.03%	0.00%	0.03%	0.00%	0%
5UTR	0.084%	0.00%	0.07%	0.02%	0.00%	0.02%	0.00%	0.00%	0.00%	0.00%	0%
ncRNA	0.228%	0.00%	0.20%	0.02%	0.08%	0.05%	0.03%	0.03%	0.05%	0.00%	0%
pseudo	0.068%	0.00%	0.07%	0.15%	0.14%	0.17%	0.03%	0.15%	0.14%	0.00%	0%
rRNA	0.001%	12.50%	0.00%	0.22%	0.21%	0.25%	0.13%	0.18%	0.17%	0.00%	0%

	SPRI uscfDNA										
Annotation	Genome	DNaseI uscfDNA	dsDNase uscfDNA	S1 Nuclease uscfDNA	P1 Nuclease uscfDNA	Exonuclease 1 uscfDNA	dsDNA Library Kit uscfDNA	Nick Repair Enzyme uscfDNA	Heatshock+ (Control) uscfDNA	Heatshock- uscfDNA	NTC uscfDNA
Intergenic	54.573%	60.38%	81.13%	60.62%	76.69%	56.15%	66.30%	50.76%	52.52%	49.48%	54.17%
Intron	40.756%	35.52%	15.48%	33.44%	21.03%	36.46%	29.96%	40.03%	38.89%	42.23%	39.24%
Exon	1.203%	0.57%	0.35%	0.60%	0.31%	0.79%	0.37%	1.27%	1.17%	1.16%	2.08%
Promoter	1.165%	1.73%	1.76%	3.31%	1.01%	4.15%	1.83%	5.26%	4.90%	4.44%	2.08%
TTS	1.050%	0.87%	0.90%	1.21%	0.70%	1.40%	1.02%	1.38%	1.26%	1.42%	0.35%
3UTR	0.869%	0.42%	0.09%	0.39%	0.12%	0.35%	0.25%	0.75%	0.74%	0.73%	1.04%

5UTR	0.084%	0.09%	0.03%	0.00%	0.04%	0.19%	0.09%	0.30%	0.26%	0.27%	0.00%
ncRNA	0.228%	0.09%	0.09%	0.28%	0.04%	0.35%	0.12%	0.16%	0.16%	0.17%	0.00%
pseudo	0.068%	0.11%	0.07%	0.11%	0.04%	0.09%	0.07%	0.06%	0.09%	0.07%	0.00%
rRNA	0.001%	0.23%	0.10%	0.04%	0.04%	0.09%	0.00%	0.02%	0.01%	0.02%	1.04%

Table S9. Numerical values related to Figure 4C and Supplemental Figure 6.

	Genome	QiaC 25-35bp	QiaM 25-35bp	SPRI 25-35bp	QiaC 36-45bp	QiaM 36-45bp	SPRI 36-45bp	QiaC 46-55bp	QiaM 46-55bp	SPRI 46-55bp	QiaC 56-65bp	QiaM 56-65bp	SPRI 56-65bp	QiaC 66-75bp	QiaM 66-75bp	SPRI 66-75bp	QiaC 76-85bp	QiaM 76-85bp	SPRI 76-85bp	QiaC 85-100bp	QiaM 85-100bp	SPRI 85-100bp
Intergenic	54.573	54.72	48.35	56.29	54.07	44.82	50.56	57.97	39.47	49.98	66.67	51.43	55.03	71.17	61.22	58.31	83.7	57.85	54.23	82.11	66.15	57.56
Intron	40.756	33.07	42.14	39	36.24	42.9	41.25	34.49	44.41	41.08	24.69	40.41	39.25	20.72	32.2	37.5	13.04	37.19	39.88	14.63	30.77	36.97
Exon	1.203	3.15	2.38	0.33	0.78	2.03	1.25	1.45	1.97	1.16	1.23	0.45	0.6	0	1.22	0.14	0	0	0.76	0	0	0.21
Promoter	1.165	2.36	4.1	2.29	3.1	6.27	4.1	2.46	9.85	4.96	3.09	5.73	3.21	1.8	3.41	2.02	2.17	1.65	2.87	2.44	1.54	2.94
TTS	1.05	1.97	1.19	1.18	1.55	1.66	1.44	1.59	2.32	1.35	1.85	1.25	0.98	1.8	1.71	1.66	1.09	2.48	1.66	0.81	1.54	2.31
3UTR	0.869	1.18	0.92	0.57	1.36	1.11	0.85	0.43	0.94	0.85	0.62	0.27	0.54	1.8	0.24	0.14	0	0.83	0.3	0	0	0
5UTR	0.084	0	0.4	0.06	0.19	0.52	0.24	0	0.52	0.31	0	0	0.18	0	0	0	0	0	0.15	0	0	0
ncRNA	0.228	0.79	0	0.13	0.39	0.33	0.21	0	0.28	0.21	0	0.27	0.17	0	0	0.14	0	0	0.15	0	0	0
pseudo	0.068	0	0	0.08	0	0.07	0.07	0.43	0.09	0.07	0	0.18	0.02	0	0	0.07	0	0	0	0	0	0
rRNA	0.001	2.76	0.53	0.06	2.33	0.29	0.03	1.16	0.15	0.02	1.85	0	0.02	2.7	0	0	0	0	0	0	0	0
